# High Throughput Analyses of Budding Yeast ARSs Reveal New DNA Elements Capable of Conferring Centromere-Independent Plasmid Propagation

**DOI:** 10.1534/g3.116.027904

**Published:** 2016-02-08

**Authors:** Timothy Hoggard, Ivan Liachko, Cassaundra Burt, Troy Meikle, Katherine Jiang, Gheorghe Craciun, Maitreya J. Dunham, Catherine A. Fox

**Affiliations:** * Department of Biomolecular Chemistry, School of Medicine and Public Health, University of Wisconsin, Madison, Wisconsin 53706; † Department of Genome Sciences, University of Washington, Seattle, Washington 98105; ‡ Department of Mathematics, College of Letters and Science, University of Wisconsin, Madison Wisconsin 53706

**Keywords:** silencers, plasmid partitioning, DNA replication origins, deep mutational scanning

## Abstract

The ability of plasmids to propagate in *Saccharomyces cerevisiae* has been instrumental in defining eukaryotic chromosomal control elements. Stable propagation demands both plasmid replication, which requires a chromosomal replication origin (*i.e.*, an ARS), and plasmid distribution to dividing cells, which requires either a chromosomal centromere for segregation or a plasmid-partitioning element. While our knowledge of yeast ARSs and centromeres is relatively advanced, we know less about chromosomal regions that can function as plasmid partitioning elements. The Rap1 protein-binding site (RAP1) present in transcriptional silencers and telomeres of budding yeast is a known plasmid-partitioning element that functions to anchor a plasmid to the inner nuclear membrane (INM), which in turn facilitates plasmid distribution to daughter cells. This Rap1-dependent INM-anchoring also has an important chromosomal role in higher-order chromosomal structures that enhance transcriptional silencing and telomere stability. Thus, plasmid partitioning can reflect fundamental features of chromosome structure and biology, yet a systematic screen for plasmid partitioning elements has not been reported. Here, we couple deep sequencing with competitive growth experiments of a plasmid library containing thousands of short ARS fragments to identify new plasmid partitioning elements. Competitive growth experiments were performed with libraries that differed only in terms of the presence or absence of a centromere. Comparisons of the behavior of ARS fragments in the two experiments allowed us to identify sequences that were likely to drive plasmid partitioning. In addition to the silencer RAP1 site, we identified 74 new putative plasmid-partitioning motifs predicted to act as binding sites for DNA binding proteins enriched for roles in negative regulation of gene expression and G2/M-phase associated biology. These data expand our knowledge of chromosomal elements that may function in plasmid partitioning and suggest underlying biological roles shared by such elements.

Eukaryotic chromosomal DNA encodes the information necessary for establishing and maintaining proper chromosome structure and expression within the nucleus, as well as chromosome duplication and distribution to daughter cells during cell division. However, our understanding of the DNA sequences that ensure appropriate chromosome structure and inheritance is incomplete. In the model eukaryotic microbe *Saccharomyces cerevisiae*, episomal plasmids are powerful tools for defining DNA sequences that govern chromosomal functions. Notably, plasmid-based assays have helped identify and characterize *S. cerevisiae* DNA replication origins and centromeres, the DNA regions essential for chromosome duplication, and segregation during cell division, respectively (Newlon and Theis 1993; Bloom 2014)

Plasmid-based assays of telomeres and transcriptional silencers, DNA sequences that direct the formation of silent chromatin, the budding yeast version of heterochromatin, have defined a DNA element, the Rap1 protein-binding site (RAP1 site), capable of linking chromosomal regions to the inner nuclear membrane (INM) via its interactions with the silencing protein Sir4 (Kimmerly and Rine 1987; Kimmerly *et al.* 1988; Longtine *et al.* 1992, 1993; Ansari and Gartenberg 1997; Andrulis *et al.* 2002). In the chromosomal context, this juxtaposition to the INM enhances the stability of silent chromatin required for yeast mating-type identity, as well as the stability and structure of telomeres (Gotta *et al.* 1996; Andrulis *et al.* 1998; Nagai *et al.* 2010; Taddei and Gasser 2012; Towbin *et al.* 2013). On plasmids, this mechanism promotes robust centromere-independent plasmid distribution, or partitioning, to daughter cells during cell division. Thus plasmid-based studies provided the first clues about the chromosomal architectural function of Rap1. Despite this evidence that plasmid-partitioning elements have the potential to reveal fundamental features of chromosome biology, our knowledge about such elements in the genome is extremely limited due to the lack of a facile systematic assay for identifying them. In this study, we present a high throughput plasmid-based approach that exploits a recently developed suite of methods for high throughput mapping of yeast DNA replication origins (ARSs) to identify dozens of putative plasmid partitioning elements in the yeast genome (Liachko *et al.* 2013).

Plasmid replication requires that the plasmid contains a DNA replication origin, which is a specific chromosomal region where DNA replication initiates. The yeast genome contains ∼400 confirmed origins, and each of these contains a specific DNA binding site for the origin recognition complex (ORC), the protein complex that selects origins in all eukaryotes (Siow *et al.* 2012). A yeast origin can confer replication to plasmids, and this ability led to the plasmid-based ARS (Autonomously Replicating Sequence) assay (Newlon and Theis 1993). Thus, yeast chromosomal origins are named ARSs. In combination with traditional molecular cloning-based mutagenesis, the ARS assay has allowed the core DNA sequence elements required for origin function and their relative organization to be defined for several origins (Marahrens and Stillman 1992; Newlon and Theis 1993; Huang and Kowalski 1993, 1996; Rao *et al.* 1994; Theis and Newlon 1994, 1997; Crampton *et al.* 2008; Chang *et al.* 2008, 2011). A classic study used comprehensive linker-scanning mutagenesis to dissect a ∼150 bp region containing the *ARS1* (a.k.a. *ARS416*) origin and revealed four distinct modular elements, a conserved A-element and poorly conserved B-elements, B1, B2, and B3, and helped establish *ARS1* as the paradigm ARS used in many subsequent biochemical studies (Marahrens and Stillman 1992). Biochemical studies have established that the A and B1 elements together comprise the ORC binding site (Bell and Stillman 1992; Rao and Stillman 1995). The B2 element helps load the eukaryotic replicative helicase, while the B3 element binds a transcription factor that excludes nucleosomes from *ARS1* (Simpson 1990; Marahrens and Stillman 1992; Lipford and Bell 2001; Wilmes and Bell 2002). The conclusions about *ARS1* elements have been recapitulated in studies of chromosomal origin function (Marahrens and Stillman 1994). However, because of the laborious nature of the ARS assay and traditional mutagenesis methods, less than a dozen of the ∼400 confirmed yeast origins have been characterized in any detail. Thus, it remains unclear how representative an example *ARS1* is for yeast origins. This issue is important because individual origins differ considerably from one another in terms of their functional behavior in S-phase, as well as their sensitivity to transcription interference and dependence on origin binding factors or chromatin regulators (Raghuraman *et al.* 2001; Nieduszynski *et al.* 2005; Feng *et al.* 2006; Donato *et al.* 2006; Knott *et al.* 2009, 2012; Muller *et al.* 2010; Pohl *et al.* 2012; Muller and Nieduszynski 2012; Hoggard *et al.* 2013).

Recently, a suite of high throughput methods was developed to map and delineate ARSs on a genome-wide scale (“ARSseq”) and to perform deep-scanning mutagenesis of ARSs, starting with *ARS1* (“mut-ARSseq”) (Liachko *et al.* 2013). Specifically, a fragment of *ARS1* was subjected to saturation mutagenesis, cloned into a plasmid without a native ARS, and the resulting library was used to transform yeast. The yeast cells were grown competitively in liquid culture, such that the *ARS1* variants better at propagating in the population over time were enriched relative to other fragments, while weaker fragments were depleted. Deep sequencing of the entire population of plasmids during the competition revealed the changing abundance of the plasmids; multiple individual nucleotides relevant for *ARS1* function were identified in a single experiment. *ARS1* provided a convenient platform for the initial development of this method because its core sequence had already been mapped to a short fragment suitable for deep-scanning mutagenesis. To map small core sequences for many more ARSs, we developed a second method (“miniARSseq”). Briefly, sheared DNA from a comprehensive library of functional ARSs was subcloned and screened for ARS function by a similar library sequencing method as described above. This experiment delineated the A element and flanking sequences important for propagation in yeast for > 100 ARSs, providing the raw material for further dissection of these elements.

While a plasmid’s propagation in this assay will vary with how efficiently the plasmid replicates, plasmid distribution between mother and daughter cells during yeast cell division might also be expected to contribute to variant success in this assay. Partitioning of plasmids to daughter cells is aided by “active” distribution mechanisms because the geometric features of the dividing nucleus and the rapidity of mitosis favor retention of plasmids by the mother cell (Gehlen *et al.* 2011). In addition to this morpho-kinetic barrier to plasmid diffusion, an additional SAGA-mediated tethering of DNAs to the nuclear pore complex also promotes retention of centromere-lacking plasmids by the mother cell (Denoth-Lippuner *et al.* 2014). Thus, to focus on elements critical to origin function and bypass these mechanisms that favor plasmid retention in the mother cell, the traditional ARS assay uses plasmids that include a centromere. However, centromere-independent mechanisms can also contribute to efficient plasmid distribution, including centromere-like elements (CLEs) (Lefrançois *et al.* 2013). And, as mentioned above, transcriptional silencers, DNA elements that direct the assembly of silenced chromatin at the cryptic mating-type loci *HMR* and *HML*, contain ARS elements closely associated with a plasmid partitioning function mediated by the silencer RAP1 site. These and other studies provide strong evidence that an association between plasmids and the INM is a mechanism for enhancing centromere-independent plasmid distribution during cell division, but additional mechanisms may exist that reflect distinct features of chromosome biology, including mechanisms that promote intra- and interchromosome interactions (Gehlen *et al.* 2011). The hypothesis, supported by both mathematical modeling and direct experiments, is that these types of interactions help a plasmid avoid the blockade of segregating chromosomes within the dividing nucleus at the bud neck, either by traveling around it (*e.g.*, plasmid interactions with the INM) or by “hitching a ride” on a chromosome (*e.g.*, plasmid-chromosome interactions) (Gehlen *et al.* 2011). Obviously, these basic mechanisms, and possibly others as yet undefined, reflect highly relevant aspects of chromosome architecture. However, because no genome-scale examination of partitioning elements has been reported, the breadth of such potential functions across the genome is unknown.

In this study, we adapted the ARS assay to examine both replication and potential partitioning elements in budding yeast at a genomic scale (Liachko *et al.* 2013). The miniARS library is comprised of thousands of distinct 100–200 bp chromosomal fragments (*i.e.*, miniARSs), representing over 100 yeast origins. While the initial study assayed the ability to transform yeast (*i.e.*, minimal ARS function), here we generated quantitative information about plasmid propagation efficiency by subjecting the pool of transformed yeast to a competitive growth experiment coupled to deep sequencing. The data generated by this experiment defined the chromosomal regions required for maximal plasmid propagation for 112 individual ARSs in a single experiment. They also allowed us to compare the efficiency between different ARSs. Strikingly, the *HMR*-E (mini*ARS317*) and *HML*-E (mini*ARS301*) transcriptional silencers were among the most effective ARSs in the library. We performed deep mutational scanning of mini*ARS317* and miniA*RS301*, direct ARS assays and numerical simulations that all supported the conclusion that the RAP1 site-mediated partitioning intrinsic to these ARSs was important for their high efficiency in the miniARS experiment. Significantly, additional ARS assays of several nonsilencer origins revealed previously undefined putative plasmid-partitioning elements. To define such elements more precisely and address their relative frequency among yeast origins, we transferred the miniARS library to a centromere-containing plasmid and performed a new competition experiment. Computational approaches to compare the data generated from the two different miniARS experiments allowed the parsing of the replication and putative partitioning functions on multiple ARS loci. A substantial fraction of the origins represented in these miniARS libraries, ∼25%, contained regions important for plasmid propagation in the absence of a centromere. However, in the presence of a centromere, these regions are dispensable for efficient plasmid propagation. We propose that these regions enhance plasmid partitioning to the daughter cell and therefore called them “putative partitioning regions.” Motif analyses identified 74 new putative partitioning elements within these regions in 26 nonsilencer origins out of a total of 112 origins examined. Many of these motifs were predicted to bind proteins involved in negative regulation of a number of cellular processes, including filamentous growth and gene expression, suggesting underlying linkages among chromosomal elements capable of substituting for centromere function on plasmids.

## Materials and Methods

### Cloning of selected fragments into Acen- and Cen-plasmids and ARS assays

Selected fragments showing either high or low competitive fitness in the Acen-miniARS competition experiment (as diagrammed in Figure 3 and Figure 5) were subcloned into comparable plasmids either containing or lacking a centromere [pIL13 (pCF2962) and pIL22 (pCF2963), respectively], as indicated in the figures. Three independent transformed yeast colonies for each plasmid construct were assessed using standard ARS assay methods (Chang *et al.* 2011), except that instead of using agar medium in petri plates for colony growth, the wells of 96-well plates (Falcon Tissue Culture Plate, #353705) were used. This method was an adaption of “tadpoling” (Welch and Koshland 2013). For each biological replicate, at least three independent ARS assays were performed. All replicates were combined to generate the data (PLR data presented as mean of all replicates ± standard error) shown in Figure 3 and Figure 5.

### Construction of the Acen- and Cen-miniARS libraries

The generation of the Acen-miniARS library was presented previously (Liachko *et al.* 2013). Briefly, a collection of 1–∼2 kb ARS fragments recovered from a yeast ARS library representing 75% of confirmed ARSs were further digested with DNaseI and size-selected for ∼100–200 bp fragments by gel purification. These fragments were subsequently cloned into a pRS406-derived vector (pIL22). To generate a comparable Cen-miniARS library, the original Acen-miniARS library extracted from bacteria was used as a template in multiple high fidelity 15-cycle PCR using primers oCF6560 and oCF6561, which flank the insert-cloning site in pIL22. The PCR fragments were purified, pooled, and cloned into a pRS406-derived vector containing a centromere (pIL13) using the Gibson Assembly method (Clontech In-Fusion HD Cloning Kit #639649). The derivative Cen-plasmid library DNA was used to transform bacteria. For library DNA preparation that was used to transform yeast, >2 × 10^5^ *Escherichia coli* clones were directly scraped from agar plates. The Cen-miniARS and the original Acen-miniARS libraries shared 2295 distinct fragments representing 112 origins with high confidence ORC binding sites (Eaton *et al.* 2010), out of a total of 2779 fragments representing 119 origins, indicating efficient transfer of the original library into a centromere-containing plasmid.

### MiniARS and miniARSmut competition experiments

The appropriate library DNA purified from *E. coli* was transformed into yeast (MATa W303) and individual yeast transformants were pooled to generate the initial miniARS library for a competitive growth experiment. All competitions were performed in shaking liquid cultures at 30° in growth medium lacking uracil, such that the uracil selectable marker on the plasmid was demanded throughout the experiment. Approximately 10 OD of cells were used to extract DNA at the start of the competition and at set time intervals throughout the competition. The DNA was extracted and purified and used as a source of total plasmid DNA for deep sequencing as described (Liachko *et al.* 2013). For the Acen-miniARS competition, the competitive fitness of individual fragments were determined by calculating the slope that described their change in frequency within the population using 0, 3, 6, 9, 12, 15, and 25-hr time points. The Cen-miniARS library was similarly competed, except the final time point was 30 hr.

The mini*ARS317*-mut and mini*ARS301*-mut libraries were constructed using synthetic oligos from TriLink BioTechnologies. Randomly mutagenized oligos spanning mini*ARS317*-full (153 bp) or mini*ARS301*-full (135 bp) were synthesized with a mutation frequency of 1.2% and 1.8% at each position for mini*ARS317*-mut and mini*ARS301*-mut, respectively. The mutagenized oligos contained invariant 5′ and 3′ elements for subsequent primer annealing and PCR amplification. PCR products were cloned using Gibson assembly into the Acen-vector pIL22, and *E. coli* clones were pooled and used to prepare the miniARSmut libraries. The plasmid libraries were transformed into W303 yeast, and yeast clones were pooled to generate the starting miniARSmut library in yeast. Each miniARS-mut library was grown competitively in liquid batch culture as described for the Acen- and Cen-miniARS libraries above. For these ARSmut experiments, competitive fitness was defined as the ratio of a given fragments’ frequency at the end of the competition to its frequency at the start of the competition.

The plasmid DNA extracted from the yeast population were subjected to analyses by deep sequencing as described (Liachko *et al.* 2013). Sequencing data for this project are available at the NCBI Sequence Read Archive BioProject Accession #SRP065331. Fragment coordinates were compared to chromosomal coordinates (sacCer1) of confirmed yeast DNA replication origins annotated in the yeast Origin Database (Siow *et al.* 2012; http://cerevisiae.oridb.org). For all analyses in this study, only fragments from confirmed yeast origins that contained a high confidence ORC binding site (*i.e.*, an ORCACS) annotated in a previous study were included (Eaton *et al.*, 2010). The first nucleotide of the T-rich strand of the ORC binding site corresponded to the first nucleotide of the Extended ACS (EACS) consensus as previously defined (Hoggard *et al.* 2013).

### Numerical modeling

Numerical simulations were done using MATLAB (MATLAB 8.3, The MathWorks Inc., Natick, MA, 2014). The code is provided as a text file in File S1. To account for natural variations among the cells in a population, we introduced Gaussian noise for the two main parameters in the model, replication fitness, and partitioning fitness. The results of the simulations are robust with respect to different noise levels.

### Computational analyses

A consensus RAP1 site was derived in Weblogo from the RAP1 Position Specific Frequency Matrix (PFSM). A consensus sequence for the ORC site was created in Weblogo using the sequences of the 232 ORC sites annotated previously (Eaton *et al.* 2010). For the motif scanning of putative partitioning ARSs, 50 nucleotide regions, tiled every 25 nucleotides, from −50 to +175 (skipping the ORC site itself) were analyzed for matches to annotated motifs (JASPAR CORE 2014 fungi) using the Tomtom motif comparison tool in the MEME suite (http://meme-suite.org) with a P-value cut-off of 0.05. MiniARS fragments were mapped, aligned and analyzed using standard functions in LibreOffice Calc spreadsheets. All data except for numerical simulations were graphed in R. Supplemental Material Table S1 contains the complete list of motifs identified within the 26 partitioning ARSs (MacIsaac *et al* BMC Bioinformatics 2006) http://bmcbioinformatics.biomedcentral.com/articles/10.1186/1471-2105-7-113. Genotype and Phenotype Ontology: A Gene Ontology term finder algorithm was performed in the *Saccharomyces* Genome Database (SGD, http://www.yeastgenome.org) using GO process terms, demanding a P-value cutoff of 0.01. Table S2 includes the complete GO analyses. 84 Phenotype Ontologies (PO), lists of genes associated with a particular phenotype, were downloaded from SGD. The fraction of genes identified by various motif collections that belonged to the PO terms was determined and normalized to the fraction of sequence specific DNA binders (GO: 0043565). Table S3 includes the complete PO analyses.

### Data and reagent availability

Plasmids are available upon request. Table S4 contains the names and sequences of all plasmids. Sequencing data for this project are available at the NCBI Sequence Read Archive BioProject Accession #SRP065331.

## Results

### The HMR-E and HML-E transcriptional silencers generated many of the most competitive miniARS fragments

**Figure 1:**
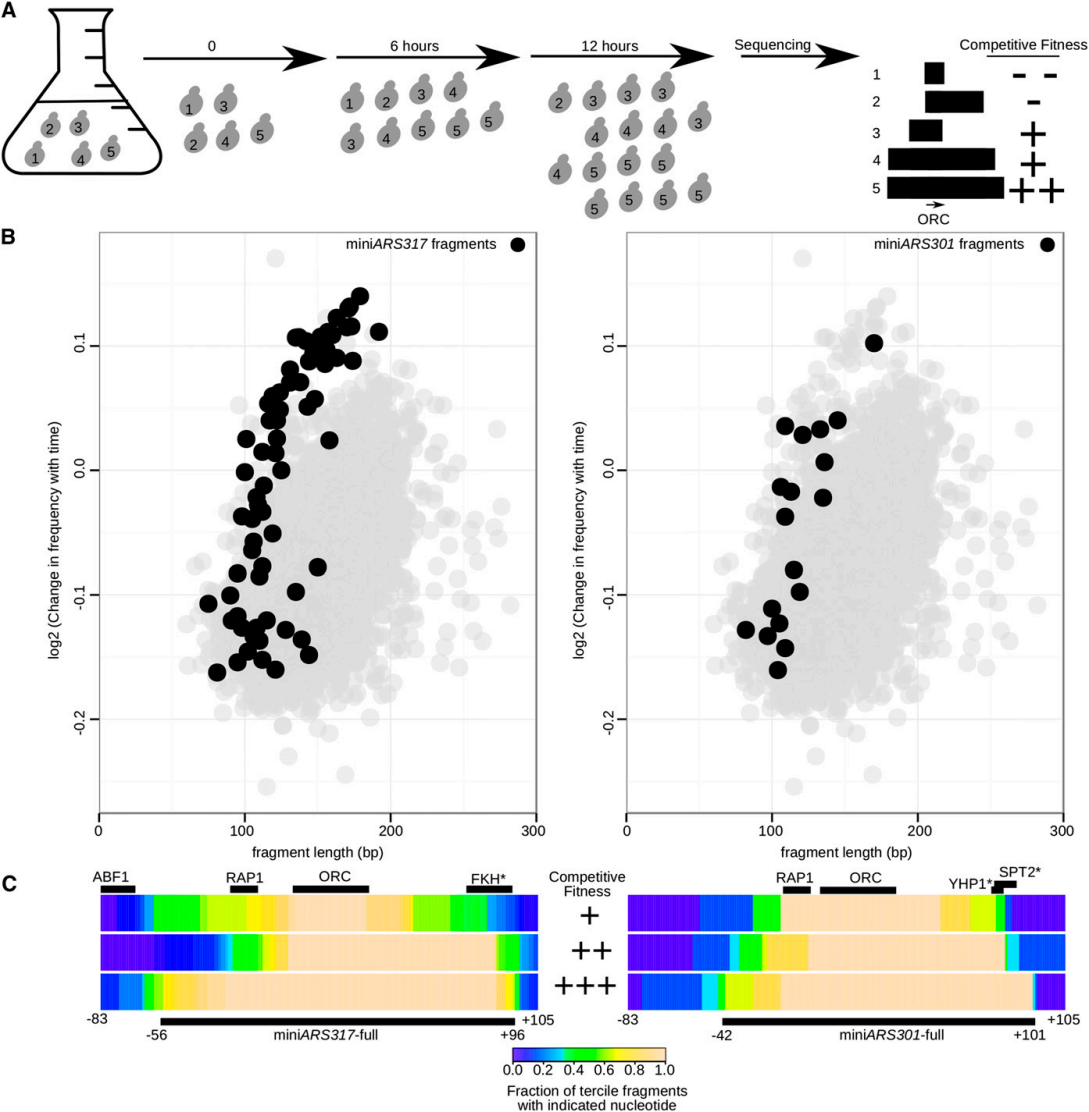
The ARSs associated with the *HMR*-E and *HML*-E transcriptional silencers generated many of the most competitive miniARS fragments in the miniARS library. (A) A pool of transformed yeast was grown competitively under conditions that selected for the plasmid. Deep sequencing measured the frequency of the miniARS fragments within the population throughout the course of the experiment. In this simple example, miniARS fragment #5 is enriched during the course of the experiment and is assigned a positive competitive fitness value. In contrast, miniARS fragment #2 is depleted during the course of the experiment and is assigned a negative competitive fitness value. If these fragments were from a single ARS (autonomously replicating sequence), ranking them based on their competitive fitness values would help indicate the minimal chromosomal region necessary for maximizing the function of this ARS. In addition, competitive fitness between origins can be compared to allow identification of the most competitive ARSs in the population. (B) The slope of the log_2_ ratios of the frequency of each miniARS fragment in the yeast cell population through the time course was used to measure each fragment’s competitive fitness (CF). This value is plotted against the length of the fragment in bp (x-axis). A gray circle designates each fragment that was assessed in this miniARS experiment. In the left panel, the *HMR*-E silencer-associated fragments (mini*ARS317*) present in the experiment are filled in black, while in the right panel the *HML*-E silencer-associated fragments (mini*ARS301*) are filled in black. (C) All of the miniARS317-fragments (n = 70, left panel) and all of the miniARS301-fragments (n = 18, right panel) were ranked based on their CF values and then divided into three distinct bins based on this value. These bins were then ranked based on their average competitive fitness (CF) values from lowest (+) to highest CF (+++). To better visualize nucleotides that were enriched in the most competitive miniARS for each silencer, an arbitrary fragment was selected encompassing each silencer and numbered relative to the first nucleotide of the T-rich strand of the ORC (origin recognition complex) binding site that was given the value “0.” Each bin was color coded as indicated to visualize the fraction of fragments within that bin that contained a given nucleotide. Thus, the most competitive bin of mini*ARS317* fragments indicated that greater than 80% of the fragments contained intact RAP1 (Rap1 protein binding site), ORC (origin recognition complex), and FKH (forkhead) sites. The most competitive miniARS silencers (miniARS317-full and miniARS301-full) used for deep mutational scanning in Figure 2 are indicated below the most competitive tercile with a thick black line. Their numbering is relative to the ORC binding site, as above. The FKH*, YHP1* (Yeast Homeo-Protein), and SPT2 (SuPpressor of Ty’s)* sites are starred to indicate that their identification is based solely on a strong motif match. In contrast, the silencer ABF1 (ARS-Binding Factor 1), RAP1, and ORC sites have been verified as such by numerous genetic and biochemical experiments.

Yeast transformed with the previously described miniARS library were grown competitively under conditions that selected for the *URA3* gene on the plasmid (Figure 1A) (Liachko *et al.* 2013). Deep sequencing of the miniARSs within the cell population at each time point revealed the composition of the miniARS library over the time course of competitive growth. Sequencing allowed the change in frequency of each individual miniARS during the competition to be determined. Each miniARS fragment could be assigned a competitive fitness value, defined as the slope of the regression line plotted through the changing fragment frequency in the population over time. Fragments enriched over time were assigned high (+) competitive fitness values, while fragments that were depleted were assigned low (−) competitive fitness values.

In Figure 1B, the competitive fitness value for each individual fragment in the miniARS experiment (*y*-axis) was plotted against the fragment’s length in base pairs (*x*-axis). Fragments overlapping selected origins were then examined for their behavior in the population (Figure 1B and Figure S1A). From these analyses, it was clear that the transcriptional silencer *HMR*-E (mini*ARS317*) generated a large number of highly competitive miniARS fragments (Figure 1B left and Figure S1D). *HML*-E associated fragments (mini*ARS301*) were also enriched among the most competitive miniARSs, though to a lesser extent than miniA*RS317* (Figure 1B, right and Figure S1D). *HMR-I* (mini*ARS318*), a weaker silencer than either of the E-silencers, was also enriched among competitive miniARSs, although to a lesser extent than either of the E-silencers (Figure S1, B–D). Thus, silencer-associated fragments were among the most competitive miniARSs and their overall competitiveness correlated with their relative strength as silencers (Figure S1D).

To define the features of *ARS317* associated with the most competitive miniARSs, the *ARS317*-associated fragments were binned into three groups based on their competitive fitness values, and the terciles were ranked from lowest (+) to highest (+++) based on their average competitive fitness value (Figure 1C). By focusing on those base pairs that were present in 80% or more of the fragments within the most competitive tercile (+++), a minimal fragment essential for maximal competitive fitness of mini*ARS317* was determined. This mini*ARS317* fragment contained base pairs −56 to +96 relative to position 0, defined as the first nucleotide of the T-rich strand of the ORC binding site (ORC site) (Figure 1C, left). This fragment will be referred to as mini*ARS317*-full. The same approach was used to map the region of mini*ARS301* required for its maximal competitive fitness (Figure 1C, right). Using the same numbering system with respect to the ORC site, this mini*ARS301* fragment contained base pairs −42 to +101 and will be referred to as mini*ARS301*-full.

This mapping revealed obvious similarities between the sequence organizations of the silencer miniARSs that generated the highest competitive fitness. In particular, both silencer miniARS-max fragments contained the silencer Rap1 binding site (RAP1 site) 5′ of the silencer ORC site. In addition, both silencer miniARSs contained sequences 3′ of this site that are not part of the defined transcriptional silencers and have no known role in silencing (McNally and Rine 1991; Rusche *et al.* 2003). Thus, both known silencer sites (RAP1, ORC) and previously uncharacterized nonsilencer sequences [*i.e.*, including putative binding sites for Fkh1/2, Yhp1, and Spt2 proteins, based on motif matches (Tomtom motif comparison tool)] were associated with mini*ARS317*-full and mini*ARS301*-full. Statistical analyses of the motifs (*e.g.*, RAP1 and FKH) tied to the function of mini*ARS317*-full are shown in Figure S2.

We performed a similar analysis with all of the fragments present in the miniARS data set combined to define an “average” ARS (Figure S3). In terms of fragment distance 3′ of the ORC site, the silencer ARSs were, in fact, quite similar to this average ARS structure, and to the structure of the paradigm ARS, *ARS1*. However, the most competitive silencer miniARSs were distinct because they contained a RAP1 site positioned 5′ of their ORC site. Given the known roles of Rap1 on silencer-containing plasmids, these data suggested that Rap1-mediated partitioning was relevant to the high competitive fitness of the silencer miniARSs in the miniARS competition experiment.

### Deep mutational scanning of miniARS317 and miniARS301

**Figure 2:**
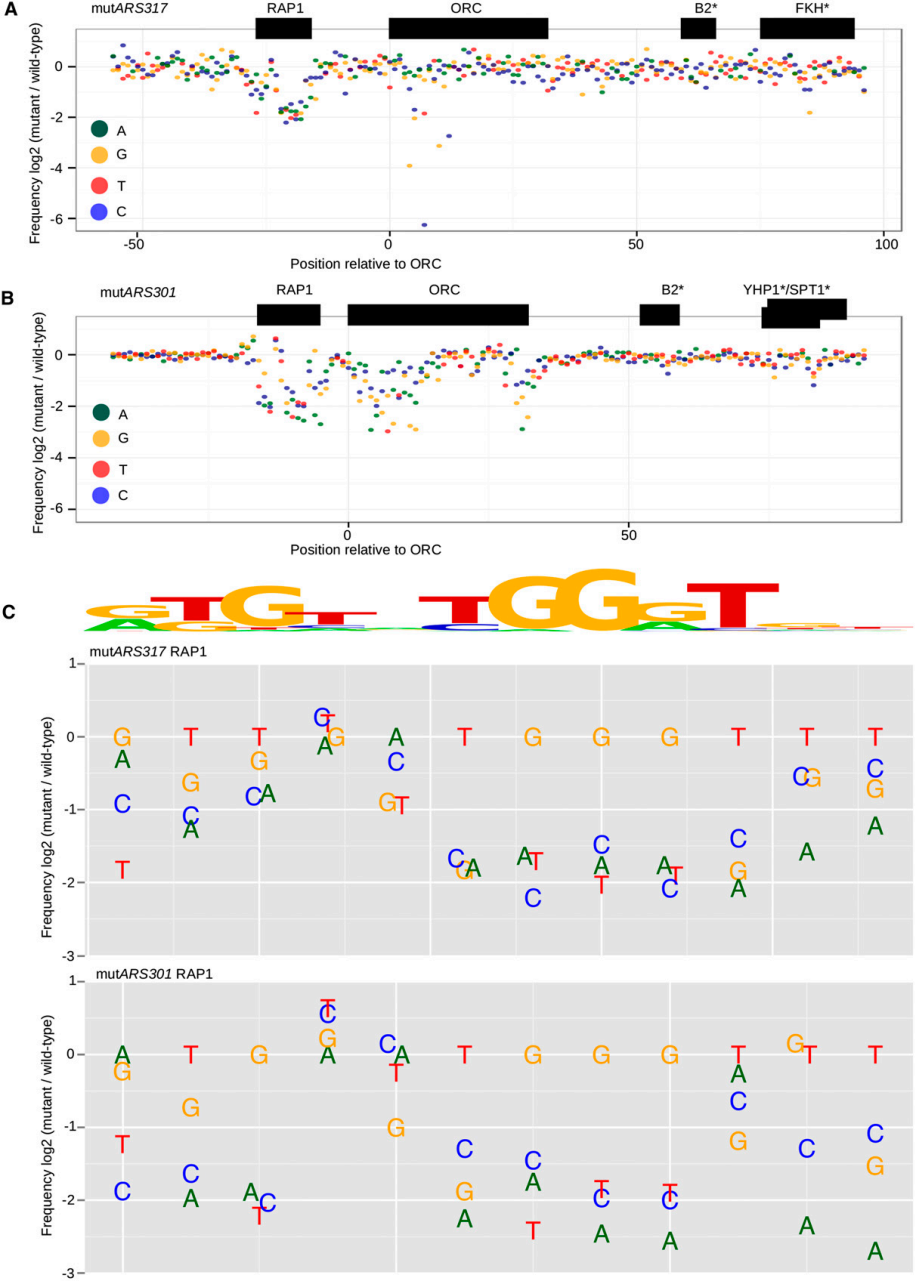
Deep mutational scanning of the *HMR*-E and *HML*-E silencer miniARSs-defined nucleotides required for their competitive fitness. (A) The data for a library of mini*ARS317*-mut fragments was graphed to indicate the individual nucleotide substitutions that affected the competitive fitness of mini*ARS317*-full. (B) As in 2A except data are from a mini*ARS301*-mut experiment. (C) Detailed analyses of the mutational profile of the RAP1 (Rap1 protein binding site) sites. A consensus RAP1 site derived from genome-wide chromatin immunoprecipitation data are shown above the profiles. FKH, forkhead; ORC, origin recognition complex; YHP1, Yeast Homeo-Protein; SPT2, SuPpressor of Ty’s.

In a previous study, we used deep mutational scanning of a mini*ARS1* fragment to query the effect of every possible single nucleotide substitution on this fragment’s competitive fitness in a single competitive growth experiment (Liachko *et al.* 2013). This strategy was applied to mini*ARS317*-full and mini*ARS301*-full to define the nucleotides most critical to their competitive fitness. Mutagenized mini*ARS317*-full (mini*ARS317-mut*) and mini*ARS301-mut* libraries were cloned into the same plasmid backbone used for the miniARS library and used to transform yeast. The yeast population was then subjected to competitive growth, and deep sequencing of the plasmid population was performed at the start and finish of the competition. The single nucleotide changes and their effects on miniARS competitive fitness for the two silencer miniARSs are summarized in Figure 2.

Several points are worth noting. First, both known (*e.g.*, ORC and RAP1) and putative (*e.g.*, FKH) sites present in the maximally competitive silencer miniARSs contained nucleotides that contributed to competitive fitness. A previous study provided evidence that the region containing the putative FKH site contains a second weaker ORC binding site (Chang *et al.* 2008). Therefore. it is possible that this region in *ARS317*max is not acting as a B3 element but instead is actually providing for additional cryptic origin activity, although based on the functional effects of this site on the competitive fitness of *ARS317*max it is much less important for *ARS317* propagation compared the silencer ORC site. Second, multiple single nucleotide changes in the known silencer RAP1 and ORC sites within both silencer miniARSs substantially reduced their competitive fitness. Third, while a small number of single nucleotide changes in the putative B2 and B3 elements of these miniARS fragments reduced their competitive fitness somewhat, the levels of reduction were less than those caused by single nucleotide mutations in either the RAP1 or ORC sites. It is possible that single nucleotide mutations were insufficient to inactivate these elements. Indeed, a B2 element consensus has been difficult to define, perhaps because it is complex and/or flexible in terms of sequence and/or position (Chang *et al.* 2011). However, it is notable that the same A–C change in their putative B2 elements reduced the competitive fitness levels of the two silencer miniARSs to similar extents.

While the importance of the ORC site to silencer miniARS competitive fitness was predicted, the distinct mutational profiles of the mini*ARS317*-full and miniARS301-full ORC sites were not. In particular, compared to the ORC site in mini*ARS301*, the ORC site in mini*ARS317* was resistant to several single nucleotide substitutions in key regions of this site, particularly in regions outside of the highly conserved A-element of the ORC site (Figure 2 and Figure S4). In Figure S3, the mutational profile of the two different ORC binding sites is presented in an expanded form to allow more detailed examination. For mini*ARS317*-full, several nucleotide positions within the conserved core A-element were unexpectedly tolerant of nucleotide substitutions that deviated from the consensus. In addition, mini*ARS317*-full was tolerant of mutations within the WTW motif, the conserved nucleotides within the B1 element of yeast origins (Chang *et al.* 2008). In contrast, for mini*ARS301*-full [and mini*ARS1* (Liachko *et al.* 2013)], the mutational profile of the ORC site followed predictions of the consensus site well. Single nucleotide substitutions throughout the core A-element and within the conserved WTW motif of the mini*ARS301* ORC site substantially reduced its competitive fitness.

In contrast to their differing ORC site mutational profiles, the silencer miniARSs’ RAP1 sites’ mutational profiles were strikingly similar (Figure 2C), suggesting that the same type of sequence-specific binding by Rap1 protein was central to maximizing the competitive fitness of both mini*ARS317*-full and mini*ARS301*-full. Thus, this high throughput miniARS-mut approach identified a known plasmid-partitioning element, the RAP1 site, as critical to the competitive fitness of both silencer miniARSs.

### Direct ARS assays showed that the silencer RAP1 site modulated plasmid partitioning without affecting intrinsic replication efficiency of miniARS317 fragments

**Figure 3:**
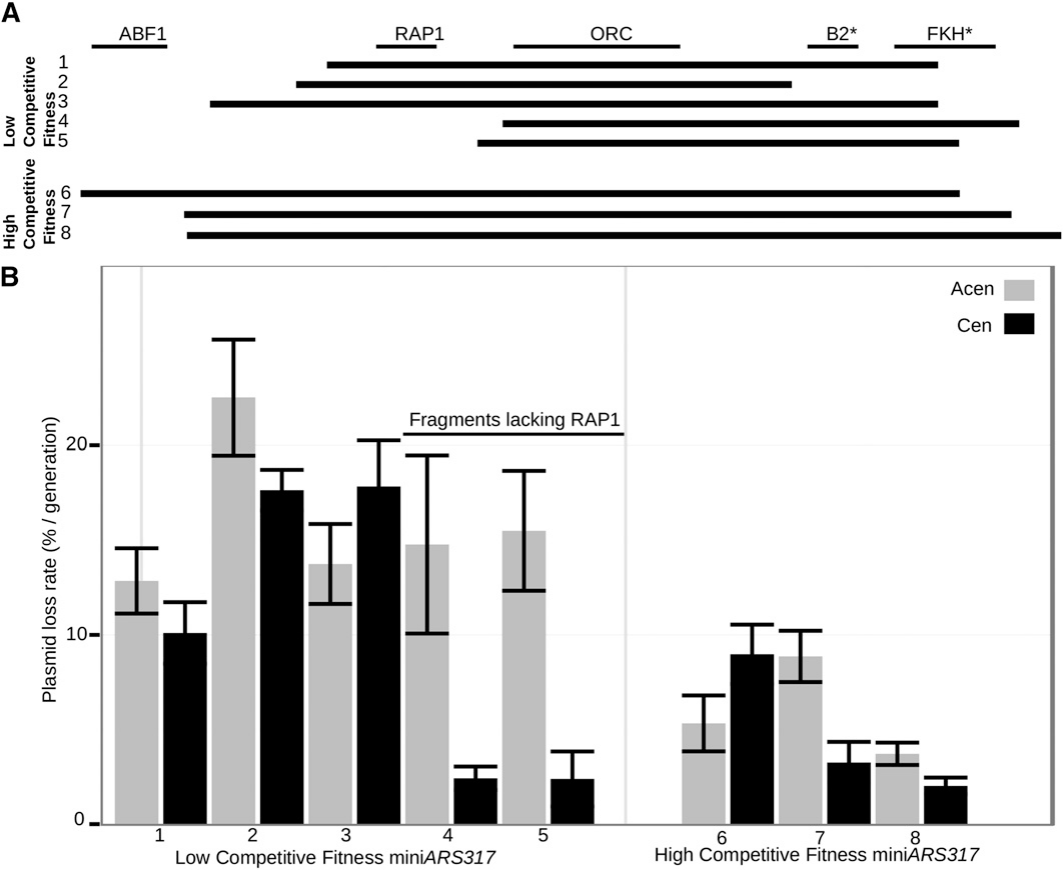
The RAP1 site in mini*ARS317* contributed to plasmid distribution but not intrinsic replication efficiency. (A) The organization and positions of key elements within silencer *ARS317* are indicated at the top by black lines. The eight thicker black lines immediately below these represent the mini*ARS317* fragment tested in direct ARS (autonomously replicating sequence) assays. Low #1–5 indicate fragments with low competitive fitness in the miniARS experiment described in Figure 1. Thus, fragment #3 lacked a portion of the putative FKH* (forkhead) site (putative B3 element), while fragment #4 contained this region of mini*ARS317* but lacked the RAP1 (Rap1 protein binding site) site. Fragments #6–8 showed high competitive fitness in the miniARS experiment. Only fragment #6 contains the silencer ABF1 (ARS-binding Factor 1) site (B) Each of the fragments represented in (A) was tested for ARS function in a centromere-containing plasmid (+centromere, black) or in an analogous plasmid lacking a centromere (–centromere, gray). Standard ARS assays were performed as described and the mean plasmid loss rate per generation (PLR) and associated standard error is indicated in the bar graph for a minimum of three independent experiments. ORC, origin recognition complex.

While the ORC site is essential for both chromosomal and plasmid origin function of silencers, there are no data to support a similar role for the silencer RAP1 site. However, several reports provide evidence that the Rap1 protein, bound to silencers or telomeres via its silencing function, contributes to plasmid partitioning (Kimmerly and Rine 1987; Kimmerly *et al.* 1988; Longtine *et al.* 1992; 1993; Ansari and Gartenberg 1997; Andrulis *et al.* 2002). These studies used standard ARS assays in which individual cells must receive at least one plasmid for a propagation event to be counted. Obviously efficient plasmid distribution is critical in such an assay. However, it was unclear how relevant partitioning was in a population-based miniARS competition experiment that assesses plasmid frequency within the entire cell population as a measure of efficiency. Furthermore, the specific silencer ARS-associated fragments described above were not directly examined in previous studies. Therefore, we directly assessed replicative *vs.* partitioning functions for several distinct mini*ARS317* fragments from the miniARS competition using standard ARS assays in plasmids that either lacked (Acen) or contained (Cen) a centromere (Figure 3A). Five of these fragments had low competitive fitness (Low #1–5), while three had high competitive fitness (High #6–8). Fragments 4 and 5 lacked the region upstream of the ORC site that contained the RAP1 binding site. If these sequences were critical for plasmid partitioning but not replication, then fragments 4 and 5 should provide for efficient ARS activity on a Cen plasmid, even though they showed low competitive fitness in the original miniARS experiment.

The ARS assays performed in the Acen plasmid used for the miniARS library revealed that the five distinct mini*ARS317* fragments with low competitive fitness values generated high plasmid loss rates (PLRs) (average PLR = 16% ± 3%), whereas the three distinct fragments with high competitive fitness values generated lower PLRs (average PLR = 6% ± 2%) (Figure 3B). These results revealed that differences in competitive fitness between these fragments determined from their behavior in the miniARS population experiment were recapitulated by a standard ARS assay in an Acen plasmid. However, ARS assays performed on the same fragments in a Cen plasmid provided evidence that the RAP1 containing region was important for plasmid partitioning but not the intrinsic replication function of mini*ARS317* fragments, as expected based on past studies. In particular, the presence of a centromere affected PLRs substantially for only the mini*ARS317* fragments that lacked the silencer RAP1 site (#4 and #5), reducing their PLRs by ∼sixfold and converting them into efficient ARSs. Analyses of variants of *ARS317*-full containing single nucleotide substitutions in the RAP1 site confirmed these results, and suggested that the RAP1 site was the only site on this fragment that was essential for Cen-independent partitioning (Figure S5). These data provided evidence that the silencer RAP1 site could at least partially substitute for the function of a centromere in an ARS assay. These data provided evidence that competitive fitness in the miniARS experiment could be modulated substantially by a fragment’s intrinsic partitioning ability.

Early studies documented that the silencers can negatively compete with centromere function when present as the sole replicators on Cen plasmids, a phenomenon named Cen-antagonism (Kimmerly and Rine 1987). If a fragment with replicator function exhibits Cen-antagonism, then it should reduce propagation of a Cen plasmid but not an Acen plasmid. While we did not rigorously test for Cen-antagonism, we note that fragment 6 in Figure 3 was ∼two-fold less stable in a Cen plasmid compared to an Acen plasmid. This particular fragment contained the entire silencer, including both the ABF1 and RAP1 sites. Hence, we suspect that this fragment associated more strongly with the Sir proteins and thus promoted a level of Sir4-Esc1-mediated partitioning that is capable of competing with (antagonizing) centromere function (Andrulis *et al.* 2002). We suspect that weaker associations of Sir proteins may be capable of promoting Cen-independent plasmid propagation without being strong enough to antagonize centromere function, such as the smaller silencer fragments we tested in Figure 3 that contained only the RAP1 site 5′ of the silencer ORC site.

### Numerical simulations revealed how the effects of cellular plasmid accumulation on replication efficiency could create a strong competitive advantage for fragments with partitioning ability in the miniARS experiment

**Figure 4:**
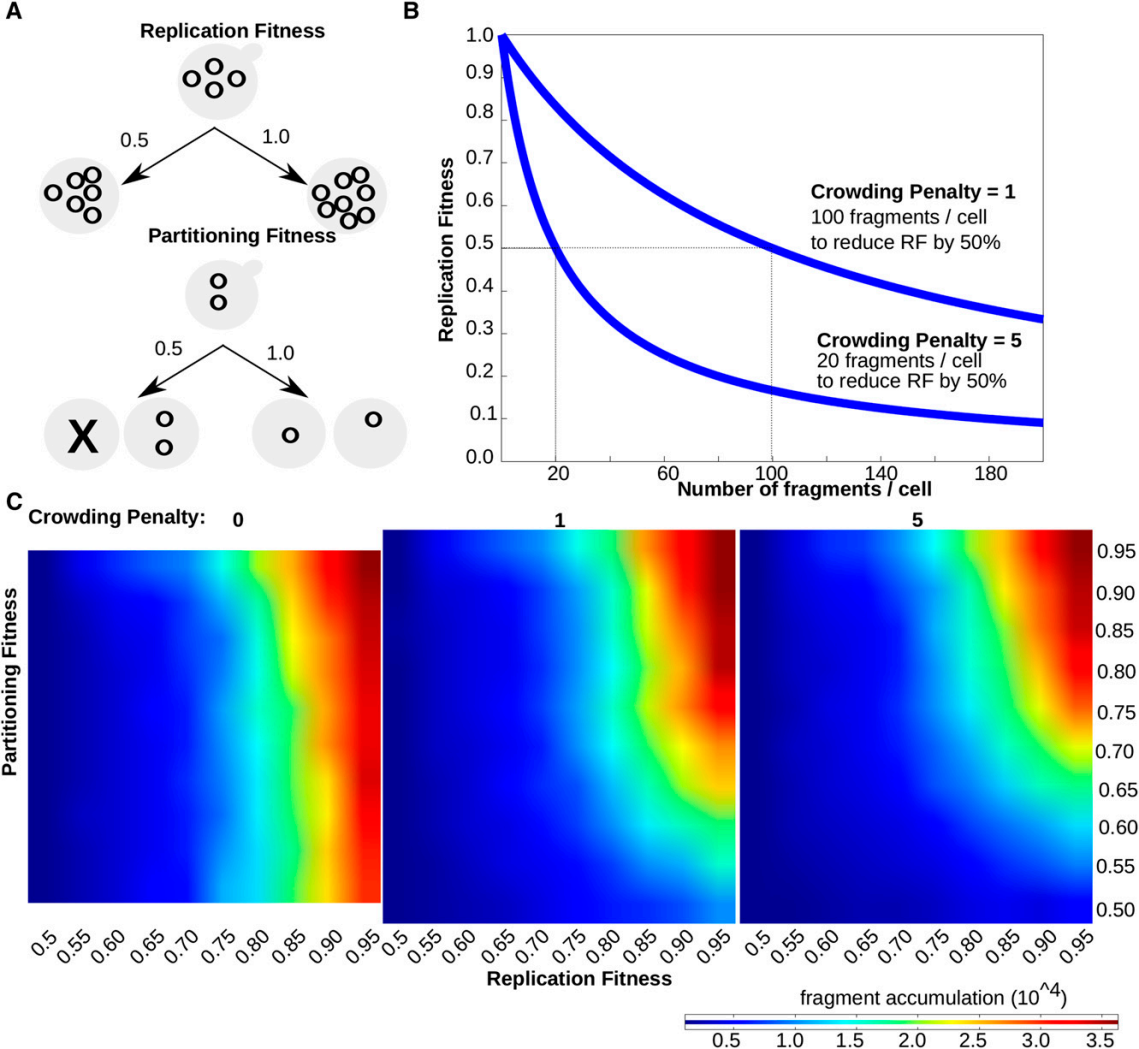
Numerical simulations revealed how differences in partitioning abilities could affect the competitive fitness of miniARS fragments. (A) Three variables were defined for these numerical simulations, as diagrammed here and discussed in the text. The Crowding Penalty was treated as a variable constant in these simulations, with all plasmids in a given simulation conferring the same Crowding Penalty value. (B) Effect of two different Crowding Penalty values on Replication Fitness and fragment accumulation per cell (C) Results of three different simulations performed under different Crowding Penalty values, as shown, depicted as heat maps. As Partitioning Fitness decreased (*i.e.*, increased probability that fragments remained in the mother cell after cell division), reduced competitive fitness resulted even if the fragment had high Replication Fitness (RF).

The Rap1-mediated mechanism that links silenced regions to the INM is considered robust. Consistent with this observation, silencer Rap1-mediated plasmid partitioning is so robust that under some circumstances it can compete against a centromere (referred to as centromere antagonism) (Kimmerly and Rine 1987). Although the data described above supported the idea that silencer Rap1-mediated partitioning could have a profound consequence on plasmid abundance in the competitive miniARS experiment, it remained unclear how plasmid partitioning might affect any particular miniARS fragment’s competitive fitness in a miniARS experiment. To address this issue, numerical simulations were performed (Figure 4). Three relevant variables were defined for these exercises as diagrammed in Figure 4, A and B: replication fitness, partitioning fitness, and crowding penalty.

Replication fitness was defined as the probability that all copies of a particular miniARS fragment present in a cell would be duplicated in a given S-phase. In the example shown, the miniARS is present in four copies in the starting cell. If the replication fitness of this fragment were 1.0, then the probability would be high that the four fragments would produce eight fragments after S-phase. A lower replication fitness of 0.5 would mean that the probability would be low that all four fragments would be copied. Partitioning fitness was defined as the probability that miniARS fragments in a cell would distribute equitably to mother and daughter cells after cell division. In the example shown, if the two fragments have a high partitioning fitness (*e.g.*, 1.0), then the probability would be high that each of the mother and daughter cells will receive a fragment after cell division. In contrast, a fragment with low partitioning fitness (*e.g.*, 0.5) would have a high probability of being retained by the mother cell after cell division. Crowding penalty was described as the probability that the replication fitness of a miniARS fragment, regardless of the intrinsic origin efficiency of the element, will decrease as the numbers of the miniARS fragments increase in a given cell. In this simulation, a crowding penalty of one means that, once a given cell has acquired 100 miniARS fragments, the replication fitness of each of those fragments is reduced by 50%. The crowding penalty was treated as a constant within each individual simulation.

In Figure 4C the results from three different numerical simulations are depicted, each under a distinct crowding penalty as indicated. In each simulation, 100 hypothetical cells each harboring a different miniARS with a distinct intrinsic replication fitness (*x*-axis) and partitioning fitness (*y*-axis) were allowed to divide for twelve generations. To account for natural variations among the cells in a population, Gaussian noise was introduced for these two parameters in the model. Importantly, the model was robust, producing similar results with different levels of Guassian noise. The predicted frequency (copy number, the measure of competitive fitness) of each miniARS fragment at the end of simulation was depicted in a heat map. In the absence of a crowding penalty (Crowding Penalty = 0), only replication fitness had a large impact on the competitive fitness of a miniARS fragment (Figure 4C). However, if the crowding penalty was increased to 1.0, differences in partitioning fitness began to substantially impact the competitive fitness of a miniARS fragment. The effect of partitioning fitness on competitive fitness becomes stronger as the crowding penalty increases. Regardless, even modest assumptions about the magnitude of a crowding penalty meant that both replication fitness and partitioning fitness could contribute substantially to the competitive fitness of a miniARS fragment. These exercises, together with data from the silencer miniARSs described above, suggested that partitioning effects in the miniARS experiment could be significant and were unlikely to be restricted to the three silencer fragments.

### Some nonsilencer miniARSs contained regions that contribute to plasmid partitioning

**Figure 5:**
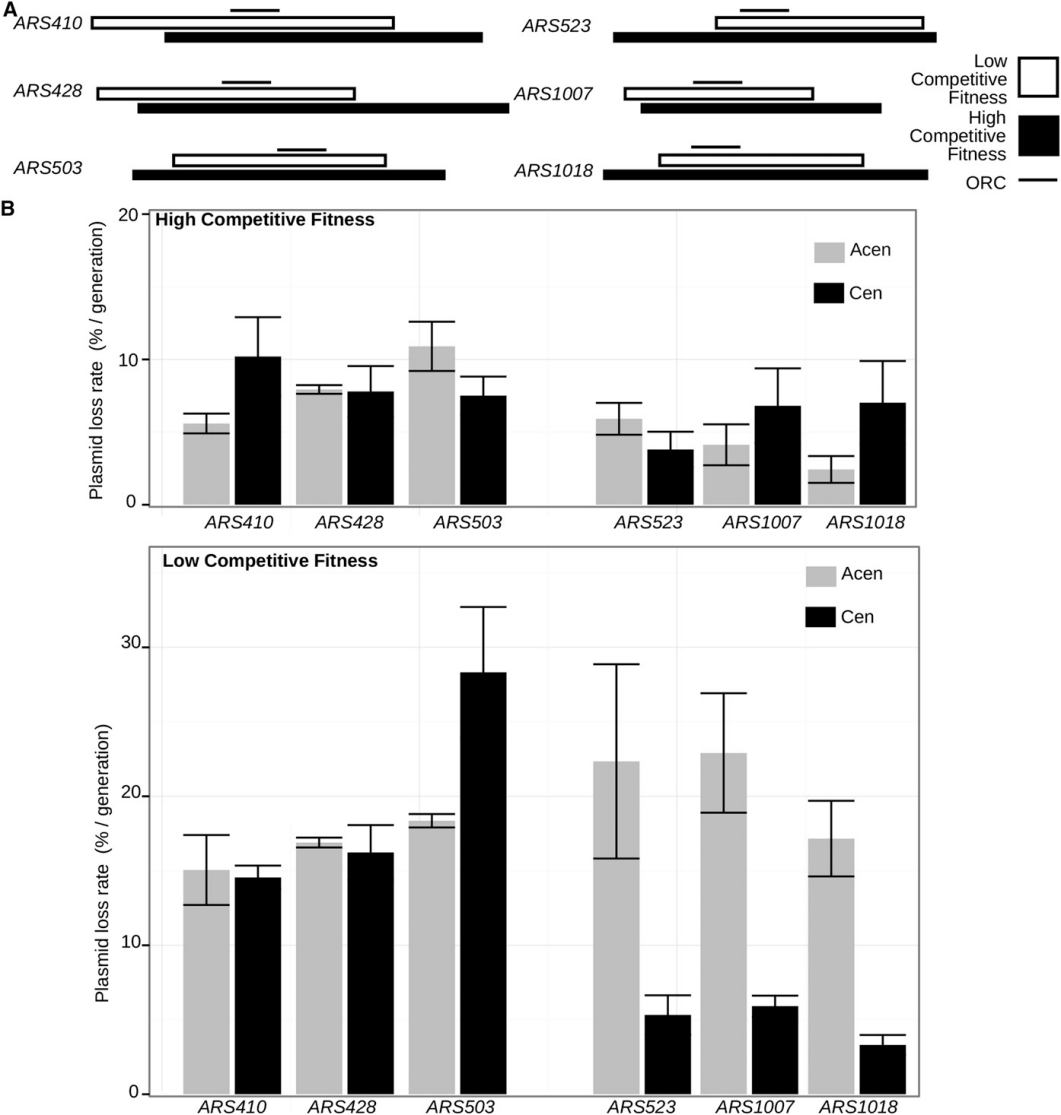
Some nonsilencer miniARSs contained regions that contributed to plasmid partitioning. (A) Diagram of low competitive (white bars) and high competitive (black bars) miniARS fragments examined by standard ARS (autonomously replicating sequence) assays in part (B). The line above each pair of fragments indicates the ORC (origin recognition complex) binding site in the standard orientation used throughout this study—the T-rich strand of the ORC site is on the top strand. (B) ARS assays were performed and presented as described for Figure 3B. Acen, lacking a centromere; Cen, containing a centromere.

Based on the data discussed thus far, the miniARS experiment was sensitive to both replication and partitioning abilities. Natural, chromosomal-based plasmid partitioning elements other than the RAP1 site have not been described. Therefore, the miniARS data set provided an opportunity to test whether other as yet undefined partitioning elements were closely associated with nonsilencer ARSs represented in the library. To test whether other ARSs might be associated with putative partitioning element(s) (*i.e.*, regions that behaved like the silencer RAP1 site), we examined fragments of low and high competitive fitness for six different nonsilencer miniARSs by standard ARS assays, as described for the mini*ARS317* experiments in Figure 4 (Figure 5A). These miniARSs were selected for further analysis because they each contained at least one fragment representative in the least and most competitive deciles within the miniARS competition, and in this way behaved similarly to mini*ARS317*.

As expected, the six distinct miniARS fragments with high competitive fitness generated relatively low plasmid loss rates (PLRs) compared to the corresponding fragments with low competitive fitness (Figure 5B). For three of these ARSs— *ARS428*, *ARS410*, and *ARS503*—the PLRs were similar for their fragments regardless of whether the plasmid contained a centromere. Thus providing a robust plasmid distribution mechanism via a centromere had no effect on ARS stability for these miniARS fragments, suggesting that their competitive fitness differences were due primarily to replication fitness differences. However, for the three remaining ARSs in this group— *ARS523*, *ARS1007*, and *ARS1018*—the fragments that produced low competitive fitness in the miniARS experiment (and high PLRs on an Acen plasmid) generated low PLRs when present on a Cen plasmid. These results suggested that the *ARS523*, *ARS1007*, and *ARS1018* fragments with low competitive fitness actually possessed intrinsically high core replication abilities, but lacked efficient partitioning abilities, behaving similarly to mini*ARS317* fragments that lacked a RAP1 site.

### Using the miniARS experiment to disambiguate partitioning and replication functions

**Figure 6:**
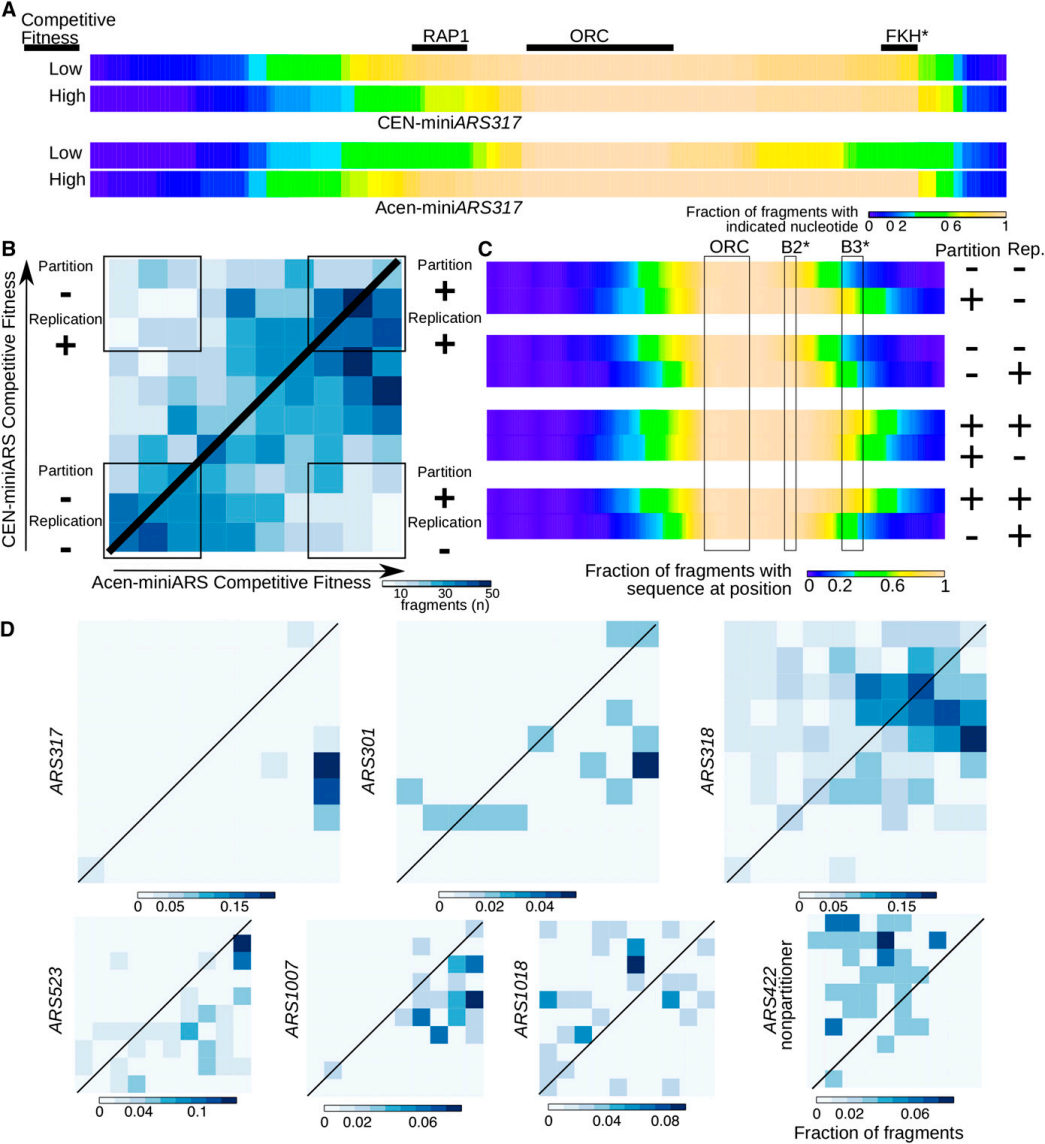
Comparing the miniARS experiments to disambiguate partitioning and replication functions associated with miniARS chromosomal fragments. (A) Mini*ARS317* fragments depleted in the Cen- (centromere containing) miniARS or the Acen- (without a centromere) miniARS experiments were compared to fragments enriched in these experiments. (B) The fragments within the shared miniARS data set (*i.e.*, fragments present in both the Acen-miniARS library and the Cen-miniARS library) that ranked at a given competitive fitness decile are indicated in this heat map. This heat map graph is divided into 100 sections, each representing a distinct combination of competitive fitness deciles within the Acen-miniARS and Cen-miniARS experiments as indicated in the figure. The bottom left corner contained fragments that performed with a low competitive fitness in both the Acen- and Cen-miniARS competitions and were therefore determined to have both weak replication and partitioning abilities relative to other fragments in the population (Partition “−”; Replication “−”). There were 288 fragments representing 62 origins in this portion of the heat map graph. The bottom right corner contained fragments that performed with a high competitive fitness in the Acen-miniARS library but a low competitive fitness in the Cen-miniARS library, and were therefore determined to have relatively strong partitioning abilities but weak replication abilities (Partition “+”; Replication “−”). There were 103 fragments representing 32 origins in this portion of the heat map graph. The top left corner contained fragments that performed with a low competitive fitness in the Acen-miniARS library but a high competitive fitness in the Cen-miniARS library, and were therefore determined to have relatively weak partitioning abilities but strong replication abilities (Partition “−”; Replication “+”). There were 105 fragments representing 33 origins in this portion of the heat map graph. (C) Combined analyses of fragments in B designed to define regions critical for maximizing partitioning ability [first (from the top) and fourth pair of heat maps] *vs.* those more critical for maximizing replication ability (second and third heat map pairs). (D) Heat map graphs as in B, except they contained only miniARS fragments from the indicated ARSs. Please see text for more details. ARS, autonomously replicating sequence; FKH, forkhead; ORC, origin recognition complex; RAP1, Rap1 protein binding site.

The data described above revealed that as yet undefined partitioning elements could influence the competitive fitness of miniARSs. To address this issue using the high throughput power of the miniARS experiment described, we transferred the miniARS library to a plasmid containing a centromere. The resulting Cen-miniARS library was used to transform yeast and a competitive growth experiment was performed as for the Acen-miniARS library. The Acen-miniARS and Cen-miniARS data sets from these competitive growth experiments were then compared (Figure 6).

If a fragment showed high competitive fitness in the Acen-miniARS experiment primarily because it contained an element(s) that performed a partitioning function, such as a RAP1 site, then it should exhibit a comparatively lower competitive fitness in the Cen-miniARS experiment because partitioning ability would no longer be an advantage. Thus, in theory, simply comparing the most competitive fragments between the two different experiments should reveal chromosomal regions that contain plasmid-partitioning element(s). For example, for mini*ARS317*, the prediction was that the RAP1 site would not be required for high competitive fitness of mini*ARS317* fragments in the Cen-miniARS experiment. To test this prediction, all of the mini*ARS317* fragments with low competitive fitness (*i.e.*, depleted over the course of competitive growth) in a given miniARS experiment were grouped and compared to all of the mini*ARS317* fragments with high competitive fitness (*i.e.*, enriched over the course of competitive growth) in the same experiment (Figure 6A). For the Cen-miniARS competition, the RAP1 site was underrepresented in the group of high competitive fitness fragments compared to the group of low competitive fitness. We interpret this to mean that the RAP1 site was not selected for in the CEN-miniARS experiment because a partitioning function provided no advantage. In contrast, and as expected from analyses in Figure 1, for the Acen-miniARS competition, the RAP1 site was overrepresented in the group of high competitive fitness compared to the group of low competitive fitness fragments. Thus, the RAP1 site was selected for in the Acen-miniARS experiment because it provided partitioning function. These data demonstrated that comparative analysis of the Acen- and Cen-miniARS competitions could be used to help identify a known partitioning element.

The comparative approach was used in a combined analysis of all miniARS fragments to determine how many miniARSs were affected substantially by an associated partitioning function in the Acen-miniARS experiment. In Figure 6B, the miniARS fragments were distributed over a heat map divided into 100 sections, each representing a distinct competitive decile in the Acen-miniARS (*x*-axis) and Cen-miniARS (*y*-axis) experiments. A perfect correlation between competitive fitness in the two different miniARS competition experiments would be expected to produce fragments only within the central diagonal portion of the graph. Such an outcome would occur if differences in partitioning bias between fragments played no role in competitive fitness, which we know from the experiments described above is not the case. Of the fragments present in the experiments, 863 (38%) fell near this diagonal line, but many other fragments fell in regions suggesting that partitioning might affect their competitive fitness. For example, 103 fragments fell within the bottom right corner of the graph (grouped in the box labeled Partition: “+”; Replication: “−”) (Note: “Replication: “−”” means only that these fragments are weak replicators relative to others in the population; any fragment that made it into the population at all had to possess some basal replicative function). These fragments showed high competitive fitness in the Acen-miniARS competition but low competitive fitness in the Cen-miniARS competition and represented 32 distinct ARSs, 28% of the ARSs analyzed in this experiment. Fragments in this region of the heat map likely contained (a) DNA sequence element(s) that contributed to partitioning fitness, giving them a high competitive fitness value in the Acen-miniARS experiment even though their intrinsic replication fitness was relatively low. Conversely, the 172 miniARS-fragments, representing 24 ARSs, within the top left corner (Partition: “−”; Replication: “+”) likely contained intrinsically strong replication ability that was masked in the Acen-miniARS experiment by being out-competed by miniARS fragments with robust partitioning ability.

To better understand the sequences that might drive partitioning *vs.* replication, we initially focused on regions that we interpreted to *enhance partitioning*. For this purpose, fragments in the bottom left corner of the graph (Partition: “−”; Replication: “−”) were grouped together and compared to fragments in the bottom right corner (Partition: “+”; Replication: “−”). These fragments should differ from each other at the level of partitioning ability. As seen in the top pair of heat maps in Figure 6C, acquisition of enhanced partitioning ability was associated with acquisition of sequences 3’ of the ORC site. Conversely, to focus primarily on regions that enhanced replication, fragments in the bottom left corner (Partition: “−”; Replication: “−”) were compared to fragments in the top left corner (Partition: “−”; Replication: “+”). These fragments should differ from each other at the level of replication ability. As seen in the second pair of heat maps in Figure 6C, acquisition of enhanced replication ability was also associated with acquisition of sequences 3’ of the ORC site, though to a lesser degree than for partitioning ability. Regardless, these analyses revealed that acquisition of either replication or partitioning ability was associated with acquisition of sequences 3’ of the ORC site, corresponding in terms of position to the B3 element in *ARS1*. Thus, at this level of combined analyses, selecting for either enhanced partitioning or replication abilities led to similar, though not identical, average fragments. A similar outcome was obtained from reverse analyses, that is examining loss of replication abilities or partitioning abilities rather than acquisition of these abilities (Figure 6C, bottom pair of heat maps). Again, both abilities tracked with sequences 3’ to the ORC binding site, but partitioning ability was more sensitive to these regions than replication. These analyses suggested that 5’ positioning of a motif relative to the ORC site, as is observed for the RAP1 site partitioning element of mini*ARS317*, is not a general prerequisite for a partitioning function. Instead, the sequence identity of particular elements is likely the larger determinant.

Heat maps analogous to that in Figure 6B were generated for individual miniARSs, including the silencer miniARSs (*ARS317*, *ARS301*, and *ARS318*) (Figure 6D). Each of these miniARSs, with the exception of *ARS422*, which served as an example of a “nonpartitioning” origin, generated, by definition, at least one fragment in the bottom right corner of the heat map in Figure 6B and thus were considered “partitioning” ARSs. The graphs for both mini*ARS317* and mini*ARS301* fragments were fairly distinct examples, in that the majority of fragments skewed toward the far right side of the graph, presumably indicative of strong partitioning capacity relative to replication capacity. In contrast, mini*ARS318*, a considerably weaker silencer than either *ARS317* or *ARS301*, generated a distinct pattern of fragments, with many falling in the upper left region of the graph, indicative of relatively strong replication capacity relative to partitioning.

Analogous heat maps for four nonsilencer miniARSs are also shown in Figure 6D. Three of these ARSs contained regions required for partitioning that could be separated from regions intrinsic to replication ability based on the ARS assays shown in Figure 5 (*ARS523*, *ARS1007*, *ARS1018*). Each of these miniARSs generated several fragments that fell on the far right side of the graph, including some in the bottom right corner, indicative of fragments containing relatively strong partitioning capacity relative to replication. In contrast, the example “nonpartitioner” mini*ARS422* did not generate any fragments that fell within the far right bottom corner of the graph. In fact, most mini*ARS422* fragments fell on the left side of the diagonal line, suggesting that many *ARS422* fragments had high intrinsic replication ability compared to other fragments in the data set, but relatively weak partitioning ability.

### Mapping regions required for plasmid partitioning

**Figure 7:**
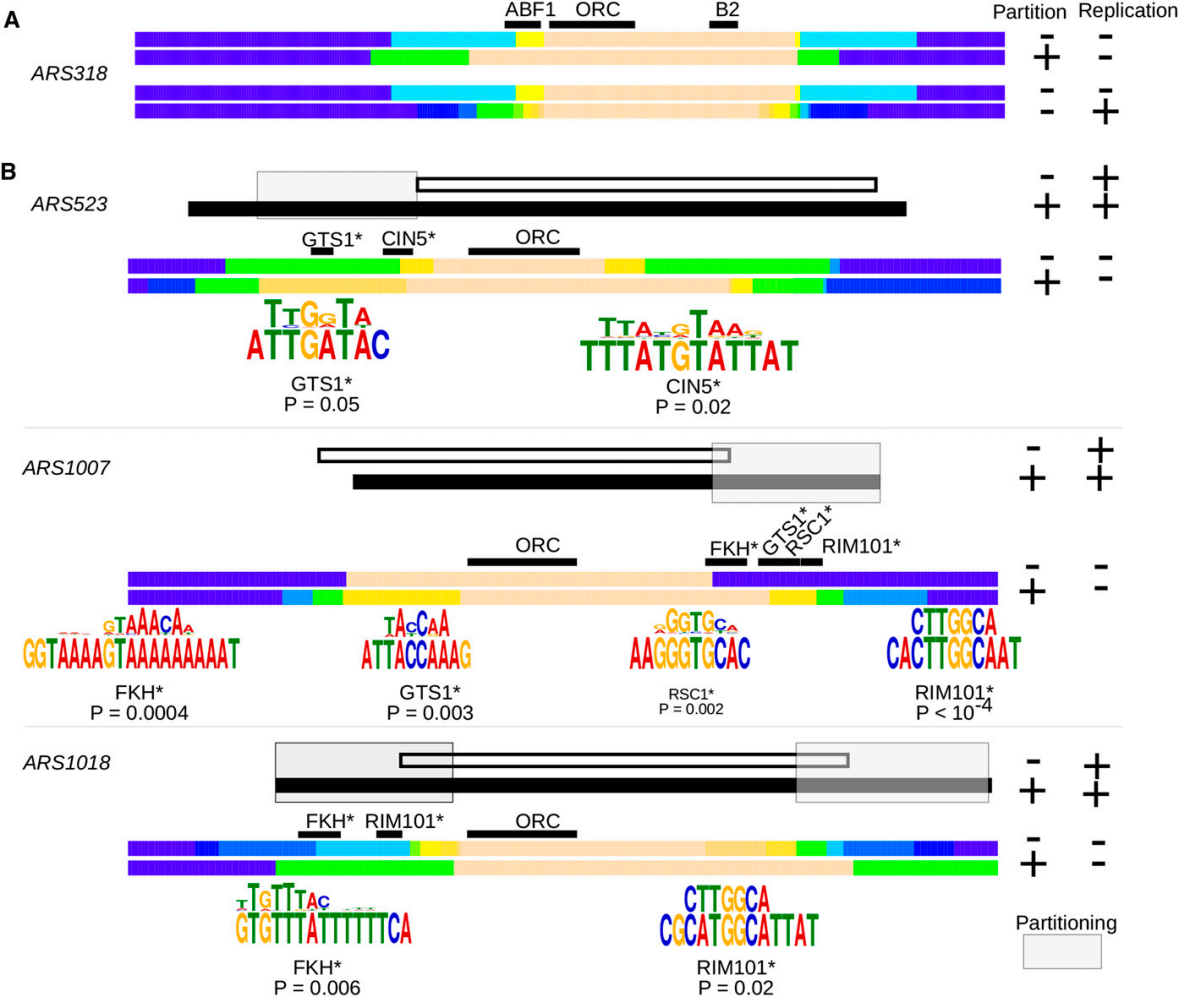
Mapping partitioning regions of miniARSs by using heat maps. (A) To map the region that provided for maximal replication fitness of mini*ARS318*, fragments that fell within the bottom left corner of the heat map graph in Figure 6D (Partition “−”; Replication “−”) were grouped and compared to the fragments that fell within the top left corner (Partition “−”; Replication “+”). To map the region that provided for partitioning of mini*ARS318*, fragments that fell within the bottom left corner of the heat map graph in Figure 6D (Partition “−”; Replication “−”) were grouped and compared to the fragments that fell within the bottom right corner (Partition “+”; Replication “−”). The color-coding for the heat maps to represent fraction of fragments with a given nucleotide is same as that used in previous figures. (B) Mapping partitioning regions of the three nonsilencer origins in Figure 5 using the same approach as for mini*ARS318*. The white and black fragments above the colored heat maps are the same low and high competitive fitness fragments from the original Acen-miniARS experiment that were assessed for ARS activity directly in Figure 5. Partitioning “−” and Replication “+” indicates the fragment that had low competitive fitness in the Acen-miniARS experiment, and thus showed a high plasmid loss rate in an Acen plasmid but a low plasmid loss rate in a Cen plasmid (white bar). Partitioning “+” and Replication “+” indicates the fragment that showed high competitive fitness in both the Acen- and Cen-miniARS experiments and thus low plasmid loss rates in both an Acen and Cen plasmid. Below these black and white fragments, the heat map graphs generated by a comparison of fragment distributions in the two different miniARS experiments (Figure 6D) for the indicated ARSs were used to map the regions acquired when the miniARS fragments gained partitioning ability, as described above and in the text. The shaded boxes indicate regions that contained putative partitioning elements based on these analyses. Motif matches (Tomtom motif comparison tool) identified within the mapped partitioning regions and their significance (P-value) are indicated. The consensus motif is shown about the actual sequence identified. The position of the ORC site, with the T-rich strand 5′ to 3′ is indicated. The binding sites for motifs are starred* to indicate that they were identified only by a motif match. Acen, without a centromere; ARS, autonomously replicating sequence; Cen, with a centromere; FKH, forkhead; ORC, origin recognition complex; RAP1, Rap1 protein binding site; ABF1, (ARS-Binding Factor 1); GTS1, Glycine Threonine Serine repeat protein; RIM101, Regulator of IME2 (Inducer of Meiosis 2); RSC1, Remodel the Structure of Chromatin; CIN5, Chromosome INstability factor 5.

Because mini*ARS318* generated many fragments distributed throughout the heat map in Figure 6D, it was used as a test case for comparing a fragment maximally fit for replication (*i.e.*, high competitive fitness in the Cen-miniARS experiment) to one maximally fit for partitioning (*i.e.*, high competitive fitness in the Acen-miniARS experiment). To map nucleotides important for the partitioning ability of mini*ARS318*, fragments from the lower left corner of the *ARS318* graph in Figure 6D were compared to fragments in the lower right corner (Figure 7A, top heat maps). The chromosomal region most enriched as mini*ARS318* acquired partitioning ability was on the 5′ side of the ORC binding site and included the silencer ABF1 site, which is essential for its silencer function. These data indicate a role for another silencer binding protein in partitioning, and are consistent with the current model that links intrinsic aspects of silencer function to plasmid partitioning (Enomoto *et al.* 1994). To map nucleotides important for maximizing the replication function of mini*ARS318*, fragments from the lower left corner of the *ARS318* graph in Figure 6D were grouped and compared to fragments in the upper left corner (Figure 7A, bottom heat maps). A distinct fragment was mapped for maximal replication efficiency that showed relatively little enrichment for nucleotides within the ABF1 site, consistent with the relatively minor role for this site in the origin function of *ARS318* compared to the region 3′ of the ORC binding site including the putative B2 element (Chang *et al.* 2008). Thus, exploiting the differential fragment distribution in the two miniARS data sets revealed distinct regions of mini*ARS318* important for replication *vs.* partitioning ability.

This mapping approach was extended to nonsilencer partitioning origins (Figure 7B and Figure S6) which had been shown by direct ARS assays to contain a region(s) required for partitioning (Figure 5). For each ARS, the region(s) predicted to contain a partitioning function were mapped as described for mini*ARS318* in Figure 7A. The fragments used in the ARS assays (Figure 5) are shown in this figure above the heat maps used to map the partitioning regions of these ARSs. From these analyses, *ARS523* contains a region 5′ of its ORC binding site required for partitioning, while *ARS1007* contains a region 3′ of its ORC binding site required for partitioning. *ARS1018* contains two different regions on either side of the ORC binding site that contribute to its partitioning ability.

We extended this mapping approach to the 23 additional nonsilencer partitioning origins that were defined as such because they contained at least one fragment within the bottom right corner of the heat map in Figure 6B (Figure S4). In the total set of 26 nonsilencer ARSs, including the three shown in Figure 7B, 15 contain regions 3′ of the ORC site predicted to contribute to partitioning, while two contain only regions 5′ of the ORC site, and nine contain regions both 5′ and 3′ of the ORC binding site. In summary, this matching approach allowed us to map new partitioning regions in 26 nonsilencer ARSs and indicated that acquisition of partitioning ability was associated with elements both 3′ and 5′ of the ORC binding site.

### Motifs within the partitioning regions defined sequence-specific DNA binding proteins enriched for particular cellular functions

Comprehensive motif analyses of the 26 nonsilencer partitioning ARSs identified in Figure 7C and Figure S4 revealed 74 discrete motifs within partitioning regions, and 26 discrete motifs within nonpartitioning regions of these ARSs (Table S1). Of the 74 putative partitioning motifs, 10 were identified in ≥ 10 of the 26 partitioning ARSs and were therefore called “high frequency” partitioning motifs (Table S1). Interestingly, while the ABF1 site was a high frequency partitioning motif, a RAP1 site was not identified in these analyses, consistent with the observation that RAP1 and ORC site juxtaposition is a defining feature of the E-silencer ARSs. Regardless, these analyses suggested that multiple different sequence-specific DNA binding proteins might influence plasmid partitioning, perhaps some as strongly as the silencer Rap1-based mechanism (*e.g.*, compare ARS assays in Figure 3 and Figure 5).

**Figure 8:**
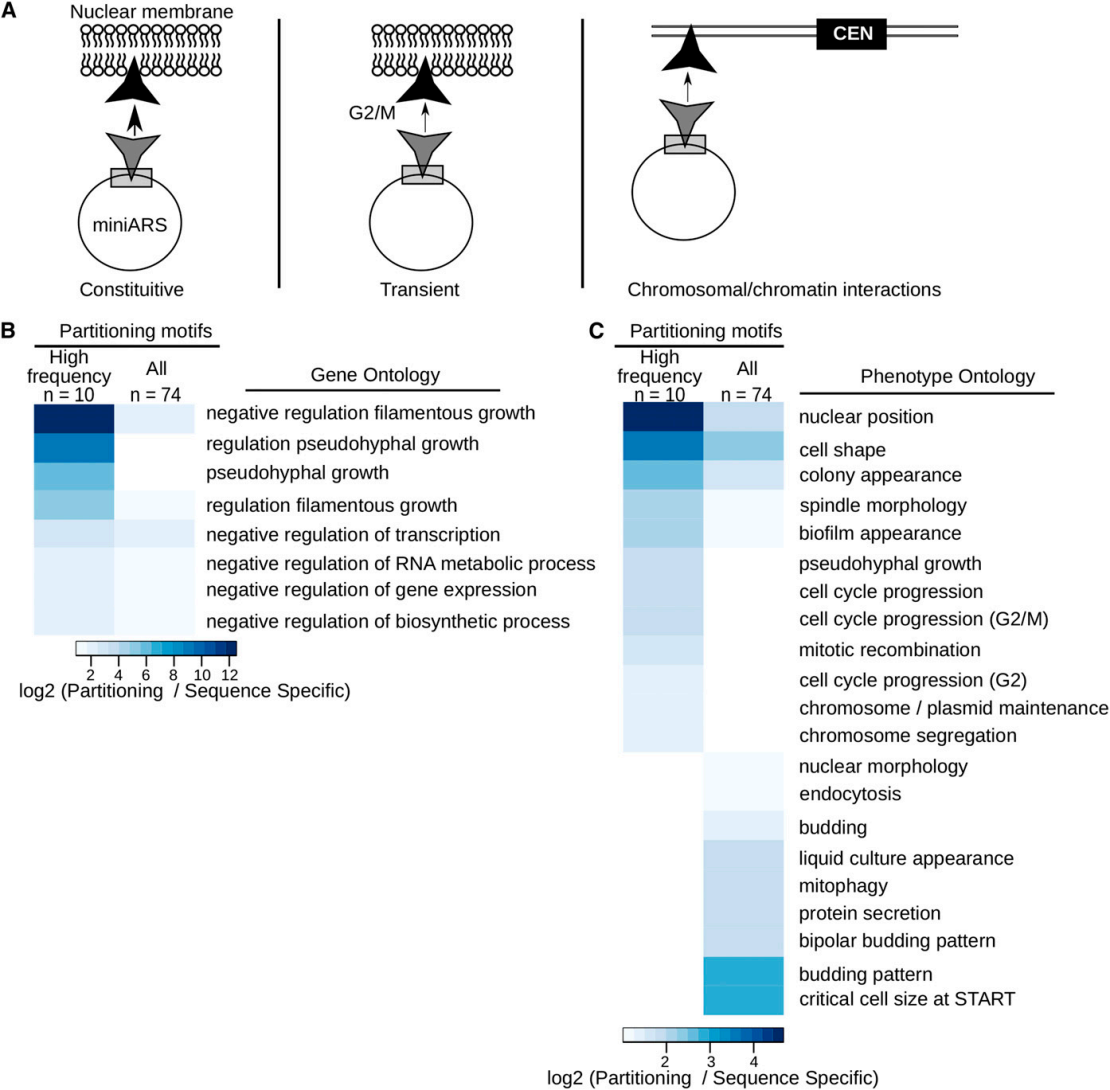
The sequence-specific DNA binding proteins identified by motifs within partitioning regions were enriched relative to all sequence-specific DNA binding proteins for particular functions, including negative regulation of RNA metabolism and growth-control strategies. (A) Depiction of mechanisms proposed to enhance Cen- (centromere) independent plasmid partitioning adapted from (Gehlen *et al.* 2011). Based on both mathematical modeling and direct experiments, a plasmid’s association with the inner nuclear membrane can enhance Cen-independent plasmid partitioning between mother and daughter cells. The silencer Rap1-mediated mechanism that tethers silencers to the inner nuclear membrane is constitutive, but cell-cycle regulated (transient) association between a plasmid and the inner nuclear membrane could also enhance plasmid partitioning, assuming that the association occurred most efficiently in M-phase as cells divide. Physical association between a plasmid and telomeres (chromosomal/chromatin interactions) also enhances Cen-independent plasmid partitioning. (B) Selected Gene Ontology (GO) term analysis (*Saccharomyces* Genome Database) of high frequency partitioning motifs [*i.e.*, motifs that were identified within at least 10 partitioning origins, n = 10 (see Table S1 and Table S2)] and all partitioning motifs (n = 74 motifs) identified within the partitioning regions of the 26 nonsilencer ARSs (autonomously replicating sequences, Figure S4). The indicated GO terms shown were enriched at least twofold and with a P-value confidence cut off of 0.01 for at least one of the collections of partitioning motifs [high frequency (n = 10) or all (n = 74)] relative to all motifs annotated as sites for sequence-specific binding DNA binding proteins (GO: 0043565, n = 256). As another control, motifs found within the partitioning ARSs but not within the mapped partitioning regions (n = 26, see Table S1, rows 3-28) were also examined. Because these motifs showed no enrichment in any GO category relative to sequence-specific DNA binding motifs (n = 256), they are not shown in the Figure. While only the GO terms associated with partitioning motifs enriched at least twofold at a P value ≥ 0.01 relative to all sequence-specific DNA binders are shown here, Table S2 lists the complete analyses of GO terms queried in these analyses. (C) A similar analysis was performed with selected Phenotypic Ontology (PO) terms. The following phenotypic categories were used in these analyses: 1, Colony appearance; 2, Chromosome and plasmid maintenance; 3, Intracellular transport; 4, Mitotic cell cycle; 5, Morphology; and 6, Development. The phenotype collections were used to annotate the motifs [high frequency partitioning motifs (n = 10), all partitioning motifs (n = 74), nonpartitioning motifs (n = 26), and all sequence-specific DNA binding proteins (n = 256; GO:0043565.)]. As in B, the enrichments for the categories are normalized to the fraction of all sequence-specific DNA binders (n = 256) with that given phenotype category. The nonpartitioning motifs showed no enrichment relative to all sequence-specific DNA binding motifs. Only PO terms associated with partitioning motifs enriched at least twofold at a P value ≥ 0.01 relative to all sequence-specific binders are shown. Table S3 lists the complete analyses of PO terms used in these analyses.

Because a large number of putative partitioning motifs were identified (n = 74) within the partitioning regions queried, we looked for shared features among the DNA binding proteins predicted to bind these motifs by using annotated data sets available on SGD. Gene Ontology (GO) analyses were performed using four different groups of sequence-specific DNA binding proteins. The first group was the entire set of sequence-specific DNA binding proteins defined on SGD (GO: 0043565, n = 256). All results from analyses of the next three selected groups were normalized to this group. The first selected group contained the DNA binding proteins predicted to bind the partitioning motifs we identified in the 26 nonsilencer origins (n = 74; “All” in Figure 8, B and C). The second selected group contained only those DNA binding proteins predicted by the high frequency partitioning motifs (n = 10; “High frequency” in Figure 8, B and C), as described above. The third selected group contained the sequence-specific DNA binding proteins, except ORC, predicted to bind motifs found within the nonpartitioning regions of the 26 partitioning ARSs, as described above. Out of the 27,522 possible GO function categories, 24 were enriched at least twofold in either the high frequency partitioning motifs (n = 10) or all partitioning motif (n = 74) groups when normalized to all sequence-specific DNA binding proteins (n = 256) (Figure 8B shows eight selected GO functions and Table S2 includes the complete analyses). Notably, the 26 nonpartitioning motifs were not enriched in any GO category relative to the 256 sequence-specific DNA binding proteins and thus are not shown in Figure 8B (Table S2). Thus, for example, DNA binding proteins defined by the high frequency partitioning motifs were enriched 12-fold over all sequence-specific DNA binding proteins in the GO category “negative regulation of filamentous growth,” while the larger group of all partitioning motifs were enriched ∼threefold in this category. All together, these analyses indicated that the putative partitioning motifs were not a random group of sequence-specific DNA binding motifs, but potentially linked to negative regulation of RNA metabolism and particular growth strategies.

We used the same strategy to assess Phenotypic Ontology terms (PO) as annotated in SGD except that, in contrast to the GO analysis, only selected phenotypes (84 in total) PO terms were queried within six general categories: mitotic cell cycle, intracellular transport, development, morphology, colony appearance, and chromosome/plasmid maintenance (see Figure 8C and Table S3 for full analysis). If a particular sequence-specific DNA binding protein is associated with a specific phenotypic category, it means that experimental data provide evidence that the wild-type function of this protein contributes to the wild-type version of this phenotype. For example, Fkh1 and Fkh2 binding sites were among the collection of high frequency partitioning motifs (n = 10). Deletions of *FKH1* and *FKH2*, while viable, alter cell cycle progression, normal cell shape and colony morphology, and other phenotypes particularly sensitive to normal G2/M regulation, and therefore cause pseudohyphal-like growth (Breeden 2000). Thus, the PO mapping provides clues about the normal biological functions controlled by the sequence-specific DNA binding proteins identified based on motif analyses. As with the GO analyses, these PO analyses indicated that the partitioning motifs identified sequence-specific DNA binding proteins enriched for particular cellular functions, suggesting connections between these functions and the ability to partition plasmids.

## Discussion

The ability of small plasmids to propagate efficiently in the model eukaryote *S. cerevisiae* has been instrumental in defining chromosomal control elements including gene promoters, DNA replication origins (a.k.a. ARSs), and centromeres. Adaptation of next-generation sequencing strategies to plasmid-based assays provides the opportunity to obtain information about these and other elements at levels of depth and breadth not previously possible. In this study, we used a competitive growth strategy coupled to high throughput sequencing to assay the competitive fitness of thousands of individual miniARS fragments simultaneously. We used this approach to try and disambiguate two fundamental contributors to efficient plasmid propagation: replication fitness and partitioning fitness. By comparing the competitive fitness behavior of fragments from a miniARS library cloned in two different plasmid backbones, one that contained a centromere (Cen) and one that lacked a centromere (Acen), we demonstrated that 26 of 112 yeast miniARS fragments contained previously undocumented putative plasmid-partitioning elements. We mapped chromosomal regions responsible for this ability and found that they contained 74 distinct motifs. Prior to this study, only one partitioning element had been defined—specifically the RAP1 site present in transcriptional silencers and telomeres. Thus, this study describes an effective high throughput approach that can help distinguish between partitioning and replication functions that influence plasmid propagation, reveals that a large fraction of ARS fragments likely contain uncharacterized mechanisms for partitioning, and identifies a large number of candidate factors that might be capable of regulating partitioning.

### A miniARS competition experiment identified silencers, ARSs known to contain a centromere-independent plasmid partitioning ability

The silencer-RAP1 site has a role in plasmid partitioning that reflects (*i.e.*, is essentially a by-product of) its chromosomal role in transcriptional silencing (Kimmerly and Rine 1987; Kimmerly *et al.* 1988; Longtine *et al.* 1992, 1993; Ansari and Gartenberg 1997; Andrulis *et al.* 2002). Specifically, data support a model where Rap1 bound to the silencer DNA interacts with Sir4, a structural component of silent chromatin itself (Moretti and Shore 2001). Sir4 in turn interacts with Esc1, a nuclear protein tightly associated with the INM (Ansari and Gartenberg 1997; Andrulis *et al.* 2002). While this series of protein–DNA and protein–protein interactions contribute to chromosomal-based transcriptional silencing, in a plasmid context it can tether a plasmid to the INM and, in doing so, provide for robust, centromere-independent plasmid partitioning.

In the Acen miniARS competition experiment, many of the most competitive miniARS fragments mapped to the Essential (E) silencers *ARS317* (*HMR*-E) and *ARS301* (*HML*-E). While it is possible that the reported “rereplication” ability of *ARS317* might contribute to this element’s exceptional behavior in the Acen miniARS competition, we propose that plasmid partitioning was likely a larger, more general factor for two reasons (Green and Li 2005; Richardson and Li 2014). First, rereplication of *ARS317* has only been observed in certain mutants, and our miniARS competitions were performed in wild type yeast. Second, silencer-mediated plasmid partitioning is well documented and could also explain the behavior of *ARS301* and *ARS318* in our studies. The silencer miniARS-mut experiments supported this conclusion by showing that the silencer’s RAP1 sites, an element known to contribute to robust plasmid partitioning, and the ORC site, an element known to be essential for plasmid replication, contributed equally to the propagation of the E-silencer ARS fragments, even though the RAP1 site is not essential for silencer-origin function. Finally, direct ARS assays confirmed that two different fragments with low competitive fitness in the Acen miniARS experiment actually possessed strong intrinsic replication efficiencies (based on their ARS activity on a Cen-containing plasmid), indicating that they lost competitive fitness in the Acen miniARS experiment when they lost Rap1-mediated plasmid partitioning ability. Therefore, the Acen mini ARS competition selected for retention of a chromosomal element known to influence plasmid partitioning.

The *HMR*-I (*ARS318*) silencer was also identified as a partitioning ARS in this study, with the partitioning ability mapping to the *HMR*-I silencer ABF1 site. Though the silencer-ABF1 site is not as recognized as the RAP1 site for its silencer-related partitioning ability, early studies of *HMR*-E indicated it could influence Cen-independent inheritance and a later study also connected it to partitioning (Kimmerly and Rine 1987; Enomoto *et al.* 1994). *HMR*-I (*ARS318*) is a weaker silencer than either *HMR*-E (*ARS317*) or *HML*-E (*ARS301*), and in this study the mini*ARS318* fragments with the highest competitive fitness value in the Acen-miniARS still showed less competitive fitness than the most competitive mini*ARS317* or mini*ARS301* fragments. The mini*ARS318* partitioning probably works through the same Sir4-Esc1-mediated mechanism, but *HMR*-I is simply less effective at binding Sir4, which would also explain why it functions as a weaker silencer.

The silencer data raised the possibility that the Acen miniARS experiment had the potential to identify other as yet unknown partitioning elements in the yeast genome and, indeed, additional direct ARS assays showed that several other ARSs represented in the library likely contained as yet uncharacterized partitioning ability. In particular, *ARS523*, *ARS1007*, and *ARS1018* each contained partitioning region(s) based on the regions’ importance in an Acen plasmid but their complete dispensability in a Cen plasmid. That is, these regions of these nonsilencer ARSs behaved like the RAP1 site within *ARS317*, in that they were not important for the intrinsic replication function of their associated ARSs. Motif analyses identified several distinct sequence motifs in the partitioning regions in these ARSs but none of these matched a RAP1 or ABF1 site, suggesting that silencer independent mechanisms for plasmid partitioning exist. Importantly, these ARSs’ partitioning regions functioned as well as the silencer *ARS317* Rap1-mediated partitioning ability, based on the PLRs determined from standard ARS assays. These locus-specific experiments, together with the silencer studies, formed the strong proof-of-principle foundation for the identification of 74 new candidate-partitioning motifs by comparing fragment competitive fitness in the Acen- and Cen-miniARS competition experiments.

Numerical simulations also supported the conclusion that partitioning ability is an important element in competitive fitness (*i.e.*, ARS efficiency) in a Acen miniARS experiment, primarily because individual yeast cells can only tolerate so many copies of a given plasmid before the replication fitness of the plasmid is reduced (expressed as a “crowding penalty”). The assumption of such a penalty is supported by recent studies that show that the large fraction of origins present in the rDNA repeats (representing up to a third of all chromosomal origins in the yeast genome, depending on rDNA copy number) functionally compete with nonrDNA chromosomal origins for limiting origin activation factors (Kwan *et al.* 2013; Yoshida *et al.* 2014). In addition, retention of plasmids in mother cells contributes to mother cell aging, another phenomenon that would contribute to the crowding penalty parameter and suppress plasmid propagation in the cell population, as measured in the miniARS experiments described here (Denoth-Lippuner *et al.* 2014). Thus, it is easy to imagine that a plasmid that fails to partition efficiently will ultimately exhibit a lower average replication fitness in an Acen miniARS competition experiment, even if it contains an intrinsically efficient replication element.

Our data provide evidence that some ARSs must have stronger partitioning abilities compared to others and that this can be highly influential to competitive fitness in the Acen miniARS experiment. Our general approach to identify nonsilencer ARSs with as yet uncharacterized plasmid partitioning functions was to compare miniARS fragment behavior in Acen miniARS and Cen miniARS experiments. Thus, we exploited the well-established function of the centromere in providing for distribution of plasmids between mother and daughter cells. However, it is important to note that recent studies show that chromosomal centromeres possess an additional function—the ability to enhance replication timing of neighboring origins—because a kinetochore protein recruits a regulatory subunit of S-phase kinase, Dbf4, thus enhancing the concentration of this kinase near centromeric origins (Pohl *et al.* 2012; Natsume *et al.* 2013). Thus, while the 26 partitioning origins were equally represented by both later and early activating chromosomal origins, suggesting no obvious bias for either type of origin in our approach, it must be acknowledged that some of the partitioning regions we mapped in our high throughput approach may also possess elements that modulate replication fitness. For example, even though partitioning ability is important for competitive fitness in the Acen miniARS experiment, any given “partitioning ARS” we have defined could, in theory, promote its partitioning via distributed elements along the fragment, and the “partitioning regions” we map may actually enhance the replication fitness in the Acen library. In this scenario, they become dispensable to high competitive fitness in the Cen library because the centromere now provides for enhanced replication fitness. This scenario is more complicated because it assumes that whatever replication enhancement is provided for by the putative partitioning region is completely redundant with the centromere’s specific mechanism. That is, the replication enhancement provided by centromeres could reasonably be predicted to “lift all boats” similarly, and therefore have no effect on relative replicative fitness differences between ARSs. Nevertheless, the recent discovery that centromeres possess a mechanism for enhancing origin activation underscores the complexity of elements and mechanisms that might modulate competitive fitness in a population-based experiment like the Acen miniARS competition. Thus, while the identification of the silencers and several lines of other published observations discussed in this study support the conclusion that plasmid partitioning regions can be identified in our high throughput approach, additional studies are essential to determine how to disambiguate “replication enhancers” (Pohl *et al.* 2013) from “core” replicative function via a high throughput approach.

### Association with the INM: a general mechanism for plasmid partitioning

The influence of INM association on plasmid behavior reflects a fundamental feature of yeast cell division (Gehlen *et al.* 2011). Because yeast cells divide by budding, the dividing nucleus and its contents are constricted at the bud neck. Thus, segregating chromosomes and their attached microtubules create a block to the free diffusion of nucleoplasmic contents, such that retention of a freely diffusible plasmid in the mother cell is a default state favored by a 9:1 ratio. This mechanism is referred to as the morpho-kinetic contribution to Acen plasmid retention in mother cells. This mechanism accounts for the majority of Acen plasmid retention, although a more active SAGA-dependent mechanism has recently been described that is substantial enough to contribute substantially to mother cell aging (Denoth-Lippuner *et al.* 2014), which would enhance the effect of the crowding penalty for any poorly partitioning Acen plasmid in the Acen miniARS experiment. Regardless, artificially anchoring a plasmid to the INM enhances plasmid inheritance substantially without affecting plasmid copy number (*i.e.*, replication efficiency) (Gehlen *et al.* 2011). Mathematical modeling explains this phenomenon by showing that movement along the outer edge of the dividing nucleus (*i.e.*, through the lipid bilayer that constitutes the INM) bypasses the blockade at the constricted bud neck and enhances a plasmid’s ability to get into the daughter cell. Thus, anchoring a plasmid to the INM provides an active mechanism to promote plasmid distribution to the daughter cell.

While the silencer Rap1-INM tethering mechanism is part of a distinct, well-studied mechanism associated with a specific type of transcriptional repression, many links between transcriptional repression and the INM have been observed in multiple model organisms (Zhao *et al.* 2009; Taddei and Gasser 2012; Towbin *et al.* 2013). Thus, it was notable that we observed an enrichment of sequence specific DNA binding proteins predicted to bind motifs within partitioning regions in the GO category “negative regulation of transcription” and related GO categories. INM association may also be a general mechanism to optimize the conditional regulation of transcription—facilitating efficient conversion from the repressed to the activated state (Green *et al.* 2012). In this regard, among the high frequency partitioning motifs identified here, several were predicted to bind proteins required for conditional transcription, including Fkh1, Fkh2, Rlm1, Dig1, and Yap6 (Watanabe *et al.* 1995, 1997; Cook *et al.* 1996; Dodou and Treisman 1997; Breeden 2000; Voth *et al.* 2007; Hanlon *et al.* 2011). Fkh1 and Fkh2 proteins activate transcription of *CLB2*-cluster genes in mid to late S-phase, but at other stages may repress or “antiactivate” target genes (Voth *et al.* 2007). Thus, perhaps some Fkh1- and Fkh2- bound chromosomal regions become associated with the INM during G2- M-phase as part of the transcriptionally repressed state. INM-anchoring-mediated plasmid partitioning would be most critical during M-phase, when the nucleus is actually dividing (Gehlen *et al.* 2011). Such a Fkh-dependent INM association may also relate to the origin-clustering function reported for the Fkh proteins (Knott *et al.* 2012). Regardless of the specific mechanism, enrichment of putative partitioning factors in the GO category “negative regulation of transcription” and PO categories related to G2- M-phase regulation may reflect links between these factors and the INM. In addition, several putative partitioning factors identified by high frequency partitioning motifs, including Azf1, Rim1, Dig1, and Yap6, physically interact with karyopherins and/or membrane proteins, suggesting links between these gene-regulatory proteins and the INM (Ito *et al.* 2001; Gavin *et al.* 2002; Ptacek *et al.* 2005; Krogan *et al.* 2006; Yu *et al.* 2008; Tonikian *et al.* 2009; Breitkreutz *et al.* 2010; Wang *et al.* 2012).

### Sequence-specific DNA binding proteins that normally enable chromosome–chromosome- or chromosome–kintechore/spindle-interactions could also contribute to plasmid partitioning

Linking a plasmid to the INM is only one general mechanism capable of enhancing plasmid partitioning. For example, artificially establishing an association between a plasmid and telomeres via a fusion protein also enhances plasmid partitioning (Gehlen *et al.* 2011). These and other experiments support a model in which tethering a plasmid to a segregating chromosome helps it to “hitch a ride” to the daughter cell. While most if not all sequence-specific DNA binding proteins may facilitate some level of chromosome–chromosome interactions, it is intriguing that the Fkh1 and Fkh2 proteins, identified here as high frequency partitioning factors, were recently implicated in establishing chromosome–chromosome interactions via 3D chromosome-capture assays (Knott *et al.* 2012). If such a chromosomal activity could be captured on a plasmid, it would be expected to enhance plasmid partitioning to daughter cells. In addition, chromosome-independent components of the segregation machinery at the nuclear bud neck could also be relevant to plasmid partitioning. In this regard, it is notable that the predicted high frequency putative partitioning factors Dig1, Fkh1, and Yap6 physically associate with components of the kinetochore and or/spindle machinery (Wong *et al.* 2007; Yu *et al.* 2008; Akiyoshi *et al.* 2010; Wang *et al.* 2012).

A deeper understanding of the mechanisms that modulate plasmid partitioning in yeast may provide novel insights into the cellular activities they reflect. Indeed, silencer-mediated partitioning has provided important tools and insights into the relationships between heterochromatin, telomere structure, and chromosomal architecture in the nucleus. An intriguing hypothesis concerning plasmid cell retention in mother cells is that mechanisms that favor this retention may act as a form of “genomic immunity,” which discourages the propagation of foreign DNA in the cell population (Denoth-Lippuner *et al.* 2014). The cost to the mother cell may be enhanced replicative aging and thus irrelevance in the population—the mother’s kill for her brood. Nonchromosomal DNA—foreign or otherwise—capable of enhancing its partitioning to daughter cells, exploiting any one of the possible mechanisms discussed above, can effectively compete for genetic influence on the cell population. While the focus in this study is yeast, the concepts are relevant well beyond this popular model organism. For example, many latent mammalian viruses exploit interactions with cellular host proteins to effectively propagate (Botchan 2004; You *et al.* 2004). In terms of designing synthetic chromosomes, it will important to learn what effects chromosomal tethering to the INM or other chromosomes might have on chromosome stability. In summary, this study presents a robust methodological framework and new data for future examination of the DNA elements and cognate binding proteins that modulate plasmid inheritance, and ultimately the chromosomal roles this function reflects.

## 

## Supplementary Material

Supporting Information
