# Supplementary material for: High Throughput Analyses of Budding Yeast ARSs Reveal New DNA Elements Capable of Conferring Centromere-Independent Plasmid Propagation
Source: G3 (Bethesda). 2016 Feb 8;6(4):993–1012. doi: 10.1534/g3.116.027904 (PMC4825667; doi:10.1534/g3.116.027904)
Supplement: Supporting Information [file supp_6_4_993__index.html]

High Throughput Analyses of Budding Yeast ARSs Reveal New DNA Elements Capable of Conferring Centromere-Independent Plasmid Propagation — Supporting Information 

# High Throughput Analyses of Budding Yeast ARSs Reveal New DNA Elements Capable of Conferring Centromere-Independent Plasmid Propagation

## Supporting Information for Hoggard *et al.*, 2016

**Files in this Data Supplement:**

- Figure S1 - Transcriptional silencers as well as some non-silencer origins generated competitive miniARS fragments in the miniARS experiment: (A) Graphs as in Figure 1A except the fragments associated with the indicated ARSs are black. (.pdf, 255 KB)
- Figure S2 - Statistical analyses of the relevance of various regions in mini*ARS317* to its competitive fitness in the miniARS competition: mini*ARS317* fragments present in the miniARS competition were grouped based on whether they contained the region that included the indicated motifs. (.pdf, 87 KB)
- Figure S3 - Combined analyses of all 2779 fragments present in the miniARS competitive growth experiment to derive the ?average? miniARS structure associated with maximal competitive fitness: The fragments were ranked based on their competitive fitness values and then divided into ten bins. (.pdf, 169 KB)
- Figure S4 - The mutational profile of the ORC binding sites within the silencer miniARSs: A consensus ORC site derived from high-confidence ORC binding sites within the yeast genome (n~232) is shown above the two profiles. (.pdf, 81 KB)
- Figure S5 - Single-nucleotide substitutions reduce the stability of an Acen plasmid but not a Cen plasmid harboring miniARS317max (see Figure 1C). (.pdf, 105 KB)
- Figure S6 - Partitioning regions were mapped for the remaining non-silencer partitioning origins identified based on the fragment distribution analyses in Figure 6B and further analyses of fragments from the individual ARSs indicated as in Figure 7B. (.pdf, 279 KB)
- File S1 - Numerical simulations of plasmid partitioning elements in budding yeast ARSs (.pdf, 18 KB)
- Table S1 - Motifs identified within the partitioning regions of non-silencer origins. (.xlsx, 53 KB)
- Table S2 - Gene Ontology (GO) analyses of the motifs identified in the 26 Partitioning ARSs. (.xlsx, 76 KB)
- Table S3 - Phenotype Ontology (PO) analyses of the motifs identified in the 26 Partitioning ARSs. (.xlsx, 886 KB)
- Table S4 - Plasmids and primers. (.xlsx, 85 KB)
