# Supplementary material for: High Throughput Analyses of Budding Yeast ARSs Reveal New DNA Elements Capable of Conferring Centromere-Independent Plasmid Propagation
Source: G3 (Bethesda). 2016 Feb 8;6(4):993–1012. doi: 10.1534/g3.116.027904 (PMC4825667; doi:10.1534/g3.116.027904)
Supplement: Supporting Information [file supp_g3.116.027904_FigureS1.pdf]

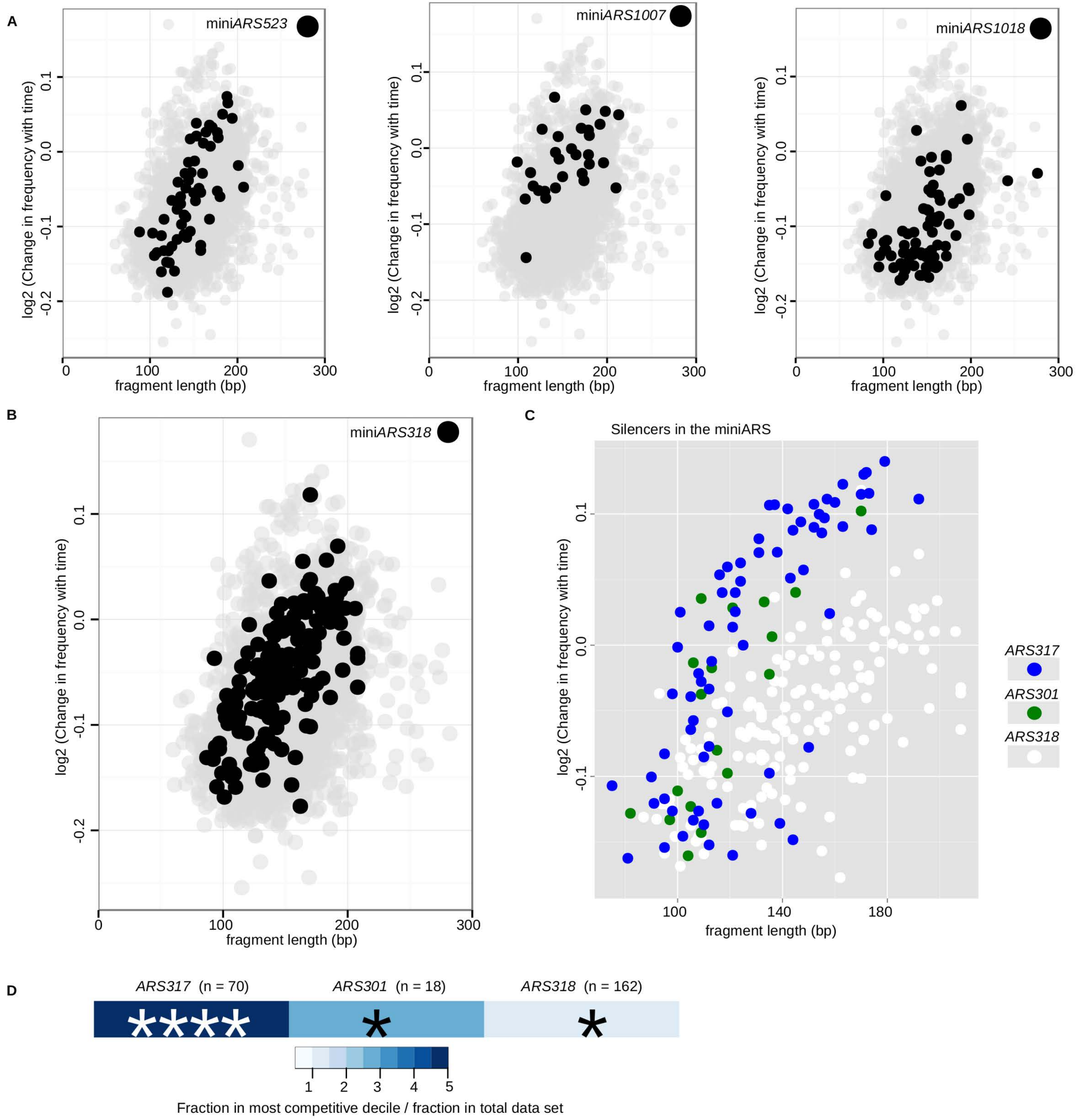
A
miniARS523
miniARS1007
miniARS1018
log2 (Change in frequency with time)
fragment length (bp)
B
miniARS318
C
Silencers in the miniARS
ARS317
ARS301
ARS318
D
ARS317 (n = 70)
ARS301 (n = 18)
ARS318 (n = 162)
****
*
*
Fraction in most competitive decile / fraction in total data set

Figure S1 Transcriptional silencers as well as some non-silencer origins generated competitive miniARS fragments in the miniARS experiment: (A) Graphs as in Figure 1A except the fragments associated with the indicated ARSs are black. (B) Graph as in (A) except HMR-I silencer (miniARS318) associated fragments are black. (C) Graph as in (A) except only the three silencer-associated fragments present in the miniARS data are shown. (D) Fragments associated with each of the three indicated silencers were enriched as assessed by the hypergeometric distribution function in the most competitive decile at the conclusion of the miniARS competition experiment relative to all fragments in the population. A * indicates a P-value of 0.01-0.05 whereas a **** indicates a P-value of 1 x 10-3-1x10-4.
