# Supplementary material for: High Throughput Analyses of Budding Yeast ARSs Reveal New DNA Elements Capable of Conferring Centromere-Independent Plasmid Propagation
Source: G3 (Bethesda). 2016 Feb 8;6(4):993–1012. doi: 10.1534/g3.116.027904 (PMC4825667; doi:10.1534/g3.116.027904)
Supplement: Supporting Information [file supp_g3.116.027904_FigureS2.pdf]

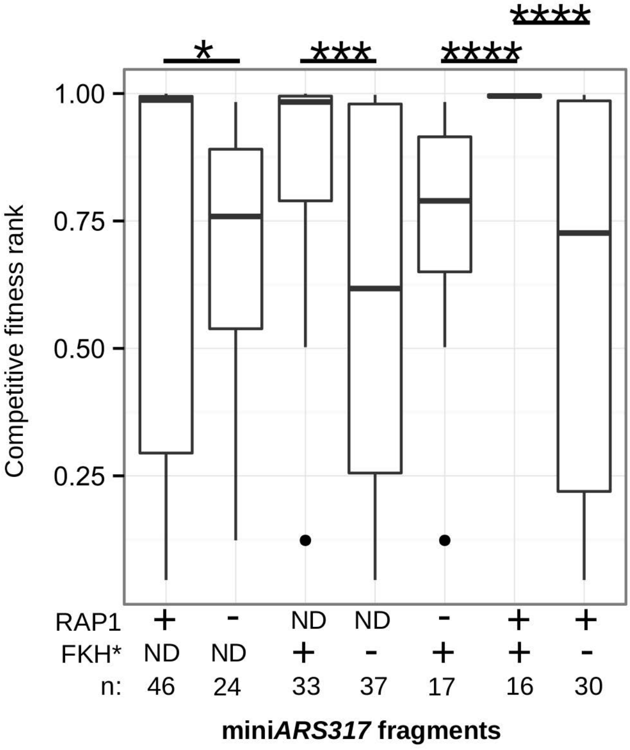
*
***
****
****
1.00
0.75
0.50
0.25
Competitive fitness rank
RAP1 + - ND ND - + +
FKH* ND ND + - + + -
n: 46 24 33 37 17 16 30
miniARS317 fragments

Figure S2 Statistical analyses of the relevance of various regions in miniARS317 to its competitive fitness in the miniARS competition: miniARS317 fragments present in the miniARS competition were grouped based on whether they contained the region that included the indicated motifs. The y-axis is the competitive fitness rank for each fragment, so that a 1.0 means a fragment between 0.75 and 1.0 ranked in the top 25% of most competitive ARS317 fragments. RAP1 of FKH* means that every fragment in that group contained the region including the RAP1 or FKH* motifs, respectively. ND means that the presence of the indicated motifs was not demanded in the indicated grouping. Thus there were 46 ARS317 fragments in the population that contained the RAP1 site, 24 fragments that lacked the RAP1 site, and 33 fragments that contained the FKH site and so on. The lines above the groups indicate what groups were compared, and about that the * symbols refer to the P-value significance of the differences between the indicated groups with P-values indicated as follows: * </= 0.05; *** </=0.0005; **** </= 0.00005.
