# Supplementary material for: High Throughput Analyses of Budding Yeast ARSs Reveal New DNA Elements Capable of Conferring Centromere-Independent Plasmid Propagation
Source: G3 (Bethesda). 2016 Feb 8;6(4):993–1012. doi: 10.1534/g3.116.027904 (PMC4825667; doi:10.1534/g3.116.027904)
Supplement: Supporting Information [file supp_g3.116.027904_FigureS3.pdf]

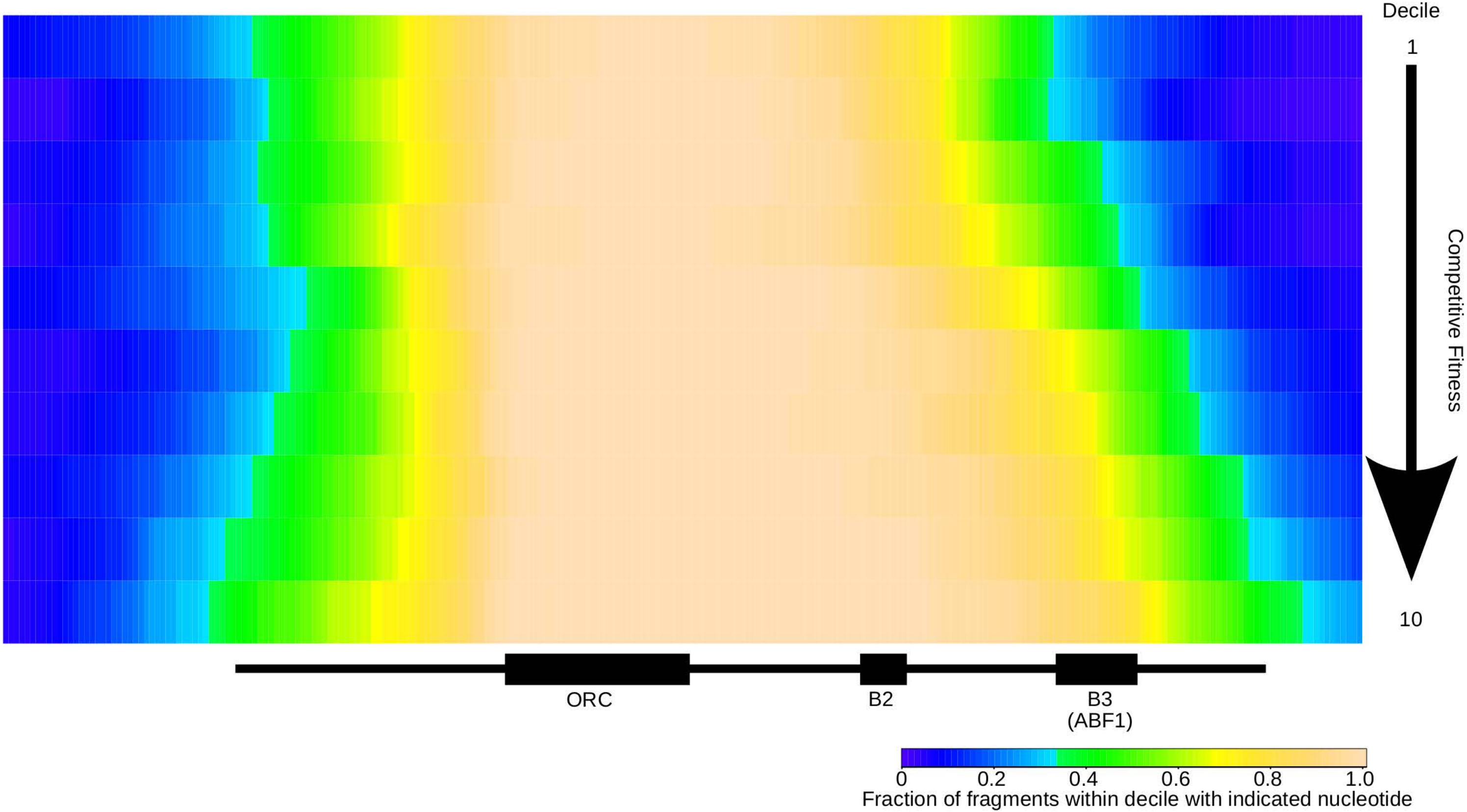
Decile
1
10
Competitive Fitness
ORC
B2
B3
(ABF1)
0
0.2
0.4
0.6
0.8
1.0
Fraction of fragments within decile with indicated nucleotide

Figure S3 Combined analyses of all 2779 fragments present in the miniARS competitive growth experiment to derive the "average" miniARS structure associated with maximal competitive fitness: The fragments were ranked based on their competitive fitness values and then divided into ten bins. Each fragment was aligned relative to position "0" on the T-rich strand of its ORC site. The bins were then ranked from their lowest to highest average competitive fitness values, and each decile color-coded as in Figure 1B to indicate the fraction of nucleotides present within each bin. The "average" competitive miniARS fragment is comprised of an ORC site and elements 3' to the to this site, including regions that correspond to the predicted B2 and B3 elements in ARS1. In ARS1, B3 is a binding site for the transcription factor Abf1, but other sequence-specific binding proteins can functionally substitute for Abf1 in ARS1, indicating that the precise identity of the protein that binds the B3 region is not critical for ARS function (Marahrens and Stillman 1992).
