# Supplementary material for: High Throughput Analyses of Budding Yeast ARSs Reveal New DNA Elements Capable of Conferring Centromere-Independent Plasmid Propagation
Source: G3 (Bethesda). 2016 Feb 8;6(4):993–1012. doi: 10.1534/g3.116.027904 (PMC4825667; doi:10.1534/g3.116.027904)
Supplement: Supporting Information [file supp_g3.116.027904_FigureS4.pdf]

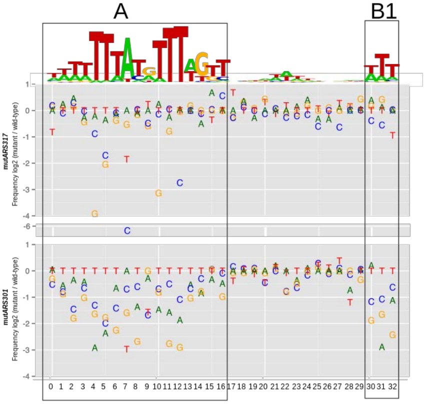

A
B1
mutARS317
Frequency log2 (mutant / wild-type)
mutARS301
Frequency log2 (mutant / wild-type)
0 1 2 3 4 5 6 7 8 9 10 11 12 13 14 15 16 17 18 19 20 21 22 23 24 25 26 27 28 29 30 31 32

Figure S4 The mutational profile of the ORC binding sites within the silencer miniARSs: A consensus ORC site derived from high-confidence ORC binding sites within the yeast genome (n~232) is shown above the two profiles.
