# Supplementary material for: High Throughput Analyses of Budding Yeast ARSs Reveal New DNA Elements Capable of Conferring Centromere-Independent Plasmid Propagation
Source: G3 (Bethesda). 2016 Feb 8;6(4):993–1012. doi: 10.1534/g3.116.027904 (PMC4825667; doi:10.1534/g3.116.027904)
Supplement: Supporting Information [file supp_g3.116.027904_FigureS5.pdf]

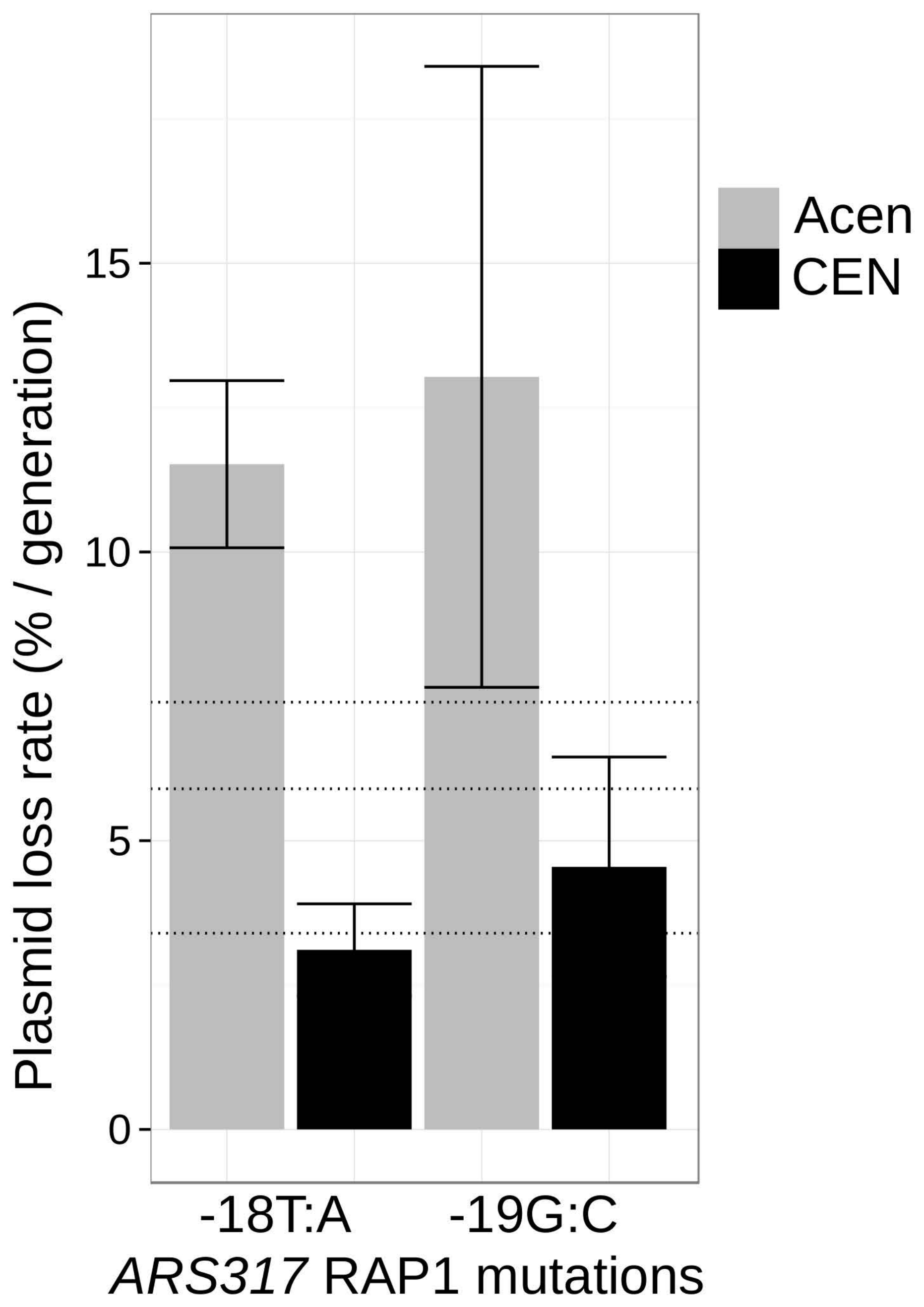

Plasmid loss rate (% / generation)
15
10
5
0
Acen
CEN
-18T:A
-19G:C
ARS317 RAP1 mutations

Figure S5 Single-nucleotide substitutions reduce the stability of an Acen plasmid but not a Cen plasmid harboring miniARS317max (see Figure 1C). ARS assays performed as in Figures 3 and 5. The dotted lines indicate the mean of the PLRs for the Acen plasmids shown in Figure 3 (fragments 6, 7 and 8) +/- one standard deviation.
