# Supplementary material for: High Throughput Analyses of Budding Yeast ARSs Reveal New DNA Elements Capable of Conferring Centromere-Independent Plasmid Propagation
Source: G3 (Bethesda). 2016 Feb 8;6(4):993–1012. doi: 10.1534/g3.116.027904 (PMC4825667; doi:10.1534/g3.116.027904)
Supplement: Supporting Information [file supp_g3.116.027904_FigureS6.pdf]

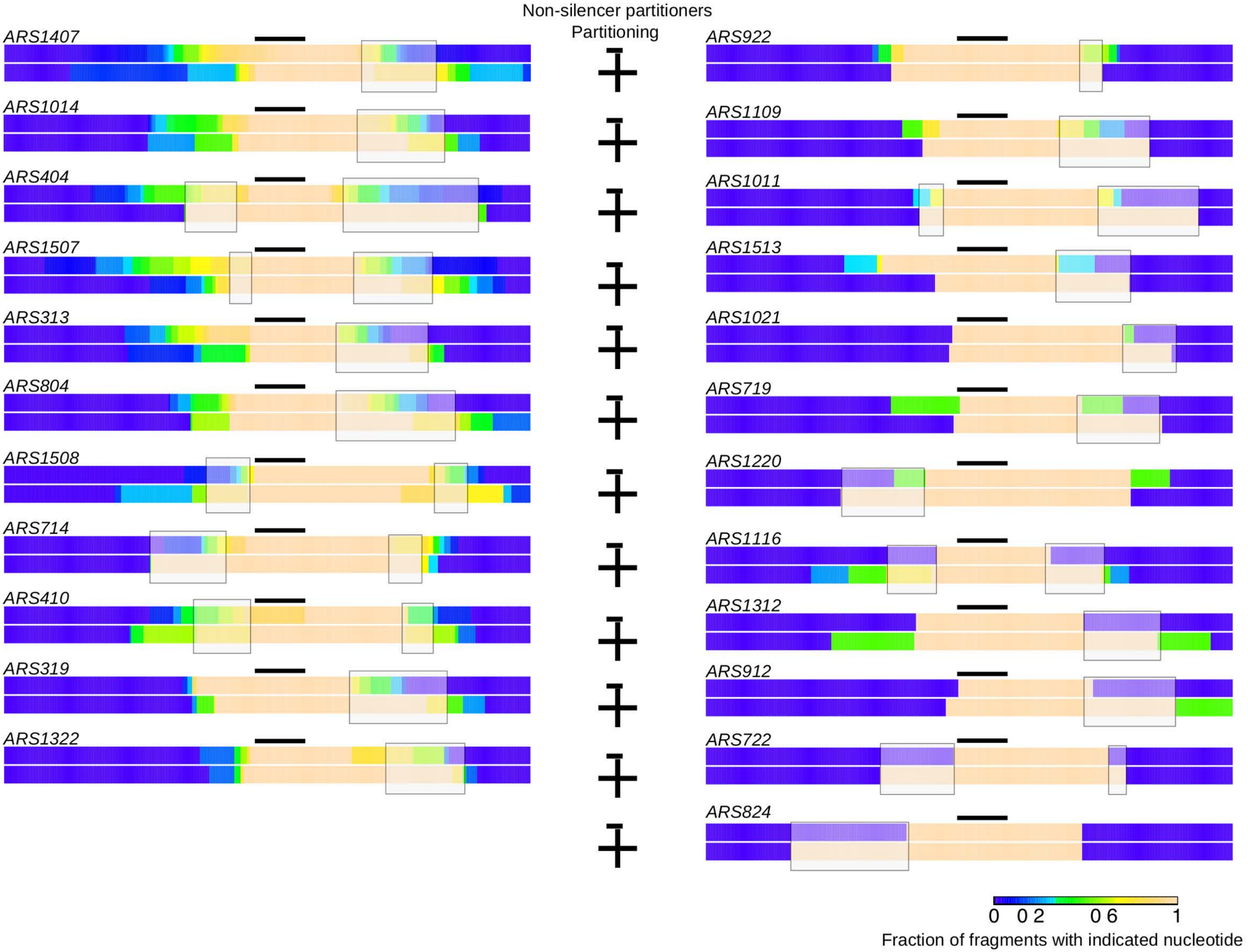
Non-silencer partitioners
Partitioning
ARS1407
ARS1014
ARS404
ARS1507
ARS313
ARS804
ARS1508
ARS714
ARS410
ARS319
ARS1322
ARS922
ARS1109
ARS1011
ARS1513
ARS1021
ARS719
ARS1220
ARS1116
ARS1312
ARS912
ARS722
ARS824
0
0 2
0 6
1
Fraction of fragments with indicated nucleotide

Figure S6 Partitioning regions were mapped for the remaining non-silencer partitioning origins identified based on the fragment distribution analyses in Figure 6B and further analyses of fragments from the individual ARSs indicated as in Figure 7B. The boxed regions indicate “partitioning regions” defined as DNA sequence gained as the fragment gained partitioning ability. Motifs identified in these regions were considered putative partitioning elements and used for the analyses in Figure 8. These motifs are listed in Table S1.
