# Supplementary material for: High Throughput Analyses of Budding Yeast ARSs Reveal New DNA Elements Capable of Conferring Centromere-Independent Plasmid Propagation
Source: G3 (Bethesda). 2016 Feb 8;6(4):993–1012. doi: 10.1534/g3.116.027904 (PMC4825667; doi:10.1534/g3.116.027904)
Supplement: Supporting Information [file supp_g3.116.027904_FileS1.pdf]

```
%%%%%%%%%%%%%%%%%%%%%%%%%%%%%%%%%%%%%%%%%%%%%%%
%                                             %
% Numerical simulations of                    %
% plasmid partitioning elements               %
% in budding yeast ARSs                       %
%                                             %
%%%%%%%%%%%%%%%%%%%%%%%%%%%%%%%%%%%%%%%%%%%%%%%



%%%% yeast cells -- ARS fragments -- simulation

%%%% rf; %% replication fitness
%%%% pf; %% partition fitness
%%%% cp; %% crowding penalty

number_of_generations = 12;


cp = 0;


number_of_cells = 0;
mean_number_of_fragments = 0;
total_number_of_fragments = 0;


for jj = 1:10 %% jj specifies replication fitness

    rf = 0.50 + 0.05*(jj-1);

    for kk = 1:10 %% kk specifies partition fitness

        pf = 0.50 + 0.05*(kk-1);



        init = zeros(10,2); %% [cell index, number of fragments]

        init(1:10,1) = 1:10; %% start with 10 cells
        init(1:10,2) = 1; %% each initial cell has exactly one ARS
fragment

        cells = init;


        %%%% simulate cell growth for given parameters rf, pf, and cp
        for gen = 1:number_of_generations
            for i = 1:size(cells,1)
                %%%% random "noise" -- variations in noise level have
```

```
very small effect
                rrf = rf*gamrnd(100,0.01); %% "gamrnd(100,0.01)" is ~
between 0.8 & 1.2
                rpf = pf*gamrnd(100,0.01); %% "gamrnd(100,0.01)" is ~
gamma(1,0.01)
                %%%% cells(i,2) = round ( cells(i,2)*(1+rrf) );
                     cells(i,2) = round ( cells(i,2)*(1+rrf* ( 1/
(1+cp*(cells(i,2)/100) ) ) ) ) ;
                separe_fragments = floor ( cells(i,2)*(1-rpf) );
                if separe_fragments > 0;
                    cells(i,2) = cells(i,2) - separe_fragments;
                    eend = size(cells,1);
                    cells(eend+1,:) = [eend+1,separe_fragments];
                end
            end
        end

        %%%% [size(cells),   mean(cells(:,2)),  sum(cells(:,2))]
        %%%% figure; plot(cells(:,1),cells(:,2),'k.');

        number_of_cells(jj,kk) = size(cells,1);
        mean_number_of_fragments(jj,kk) = mean(cells(:,2));
        total_number_of_fragments(jj,kk) = sum(cells(:,2));

    end

end

figure; imagesc(number_of_cells)
xlabel('partition fitness between 0.5 and 0.95')
ylabel('replication fitness between 0.5 and 0.95')
title('number of cells')

figure; imagesc(mean_number_of_fragments)
xlabel('partition fitness between 0.5 and 0.95')
ylabel('replication fitness between 0.5 and 0.95')
title('mean number of fragments')

figure; imagesc(total_number_of_fragments)
xlabel('partition fitness between 0.5 and 0.95')
ylabel('replication fitness between 0.5 and 0.95')
title('total number of fragments')


%%%% a few examples for "crowding penalty"
% figure;  ezplot('1/(1+5*(x/100))',[0,200,0,1])
% hold on; ezplot('1/(1+1*(x/100))',[0,200,0,1])
% hold on; ezplot('1/(1+0.2*(x/100))',[0,200,0,1])
% xlabel('number of fragments inside a cell')
% ylabel('crowding penalty')
```

```
% title('crowding penalty vs. number of fragments inside a cell')
```
